# Single‐cell transcriptomics reveals distinct cell response between acute and chronic pulmonary infection of *Pseudomonas aeruginosa*

**DOI:** 10.1002/mco2.193

**Published:** 2022-12-08

**Authors:** Xueli Hu, Mingbo Wu, Teng Ma, Yige Zhang, Chaoyu Zou, Ruihuan Wang, Yongxin Zhang, Yuan Ren, Qianqian Li, Huan Liu, Heyue Li, Taolin Wang, Xiaolong Sun, Yang Yang, Miao Tang, Xuefeng Li, Jing Li, Xiang Gao, Taiwen Li, Xikun Zhou

**Affiliations:** ^1^ State Key Laboratory of Biotherapy and Cancer Center West China Hospital Sichuan University and Collaborative Innovation Center for Biotherapy Chengdu China; ^2^ State Key Laboratory of Oral Diseases National Clinical Research Center for Oral Diseases Chinese Academy of Medical Sciences Research Unit of Oral Carcinogenesis and Management West China Hospital of Stomatology Sichuan University Chengdu China; ^3^ Department of Radiation Oncology The University of Texas MD Anderson Cancer Center Houston Texas USA; ^4^ Department of Neurosurgery and Institute of Neurosurgery State Key Laboratory of Biotherapy and Cancer Center West China Hospital West China Medical School Sichuan University and Collaborative Innovation Center for Biotherapy Chengdu China

**Keywords:** *Pseudomonas aeruginosa*, pulmonary infection, single‐cell RNA sequencing

## Abstract

Knowledge of the changes in the immune microenvironment during pulmonary bacterial acute and chronic infections is limited. The dissection of immune system may provide a basis for effective therapeutic strategies against bacterial infection. Here, we describe a single immune cell atlas of mouse lungs after acute and chronic *Pseudomonas aeruginosa* infection using single‐cell transcriptomics, multiplex immunohistochemistry, and flow cytometry. Our single‐cell transcriptomic analysis revealed large‐scale comprehensive changes in immune cell composition and high variation in cell–cell interactions after acute and chronic *P. aeruginosa* infection. Bacterial infection reprograms the genetic architecture of immune cell populations. We identified specific immune cell types, including Cxcl2^+^ B cells and interstitial macrophages, in response to acute and chronic infection, such as their proportions, distribution, and functional status. Importantly, the patterns of immune cell response are drastically different between acute and chronic infection models. The distinct molecular signatures highlight the importance of the highly dynamic cell–cell interaction process in different pathological conditions, which has not been completely revealed previously. These findings provide a comprehensive and unbiased immune cellular landscape for respiratory pathogenesis in mice, enabling further understanding of immunologic mechanisms in infection and inflammatory diseases.

## Graphical abstract

Mouse lung cell subtypes with the most significant gene expression changes in acute and chronic pulmonary infection. In the acute infection condition, activated receptors and their involved biological pathways in Cxcl2^+^ B cells, neutrophils, and inflammatory monocytes are indicated by the Kyoto Encyclopedia of Genes and Genomes (KEGG) and CellChat analysis. In contrast, signaling pathways in the Stmn1^+^ alveolar macrophages (AMs) and Stmn1^–^ AMs are illustrated by the gene set enrichment analysis. Besides, in the chronic infection condition, expansion of interstitial macrophages and related signaling pathways are shown by the KEGG analysis.

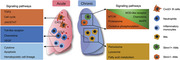

## 1 INTRODUCTION

Cell is the fundamental unit of organisms. Previous studies have shown that lung tissue is composed of more than 40 distinct cell types.^1^, ^2^, ^3^ The cumulative functions and their interactions with each other and non‐cellular components determine the physiological function and homeostasis of lung tissue. Pulmonary disease is thought to be caused by intracellular changes in lung tissue, changes in response to outer damage, intercellular communication, heterogeneity of cell types, and/or imbalance of tissue structure. Recent studies revealed a new epithelial cell subset called ionocytes in the human airway wall, which indicates that study of lung tissue still needs illumination.^4^, ^5^ There still needs more systematic and comprehensive insights into the complete cell types of the lung tissue, the cell component and function changes in the occurrence, progress and fade in the pulmonary diseases. In addition, the dynamics of molecules in health and disease state also should be illustrated. These approaches will improve our understanding of disease mechanisms, and design new diagnostic and personalized treatments.

*Pseudomonas aeruginosa* is a versatile gram‐negative pathogen with a large genome size that causes acute and chronic infections clinically in patients with compromised immune systems, especially those with bronchiectasis, chronic obstructive pulmonary disease, and cystic fibrosis (CF).^6^, ^7^, ^8^, ^9^ Pulmonary infection caused by *P. aeruginosa* is difficult to treat because of its innate and acquired resistance to antibiotics and extraordinarily intricate intracellular regulatory networks. Despite recent research insights, many aspects of the interaction between hosts and *P. aeruginosa* that can either facilitate or impair immune responses remain unknown. To understand the underlying mechanisms of the host–pathogen interaction, it is crucial to illuminate the molecular characteristics of immune cell intrinsic transcriptional changes and cell–cell interactions in acute and chronic pulmonary infections of *P. aeruginosa*.

The latest progress of single‐cell RNA sequencing (scRNA‐seq) and the concomitant calculation methods in recent years provide an extraordinary opportunity to solve the above outstanding problem.^10^, ^11^ It has been used to identify novel immune cell types, immune evasion mechanisms, and potential targets for immunotherapy in the past few years.^12^, ^13^, ^14^ In addition, previous studies of chronic diseases in model organisms and human samples have reported the diversity of cell types, including immune and stromal cells such as epithelial cells and fibroblasts.^4^, ^15^, ^16^ However, the cell states of individual cell types in the lung and immune‐related pathways in acute and chronic bacterial infection are still unknown.

Here, we performed an in‐depth characterization of immune landscapes across the lungs of healthy, acute, and chronically infected mice using single‐cell transcriptomics combined with bulk RNA‐seq, flow cytometry, and multiplex tissue imaging. This yielded extensive insights into the immune cell complexities in lung tissues and revealed the specific signatures of the molecular components in various immune cells. Specifically, excess accumulation of interstitial macrophages (IMs) is associated with the progression of chronic infection, indicating a previously unrevealed role of IMs in shaping the lung immune contexture. These data may offer opportunities for translating defined subpopulations of immune cells as diagnostic and therapeutic targets to control bacterial infectious diseases.

## 2 RESULTS

### 2.1 scRNA‐seq profiling analyzes immune cells in *P. aeruginosa*‐infected lung tissues

**FIGURE 1:**
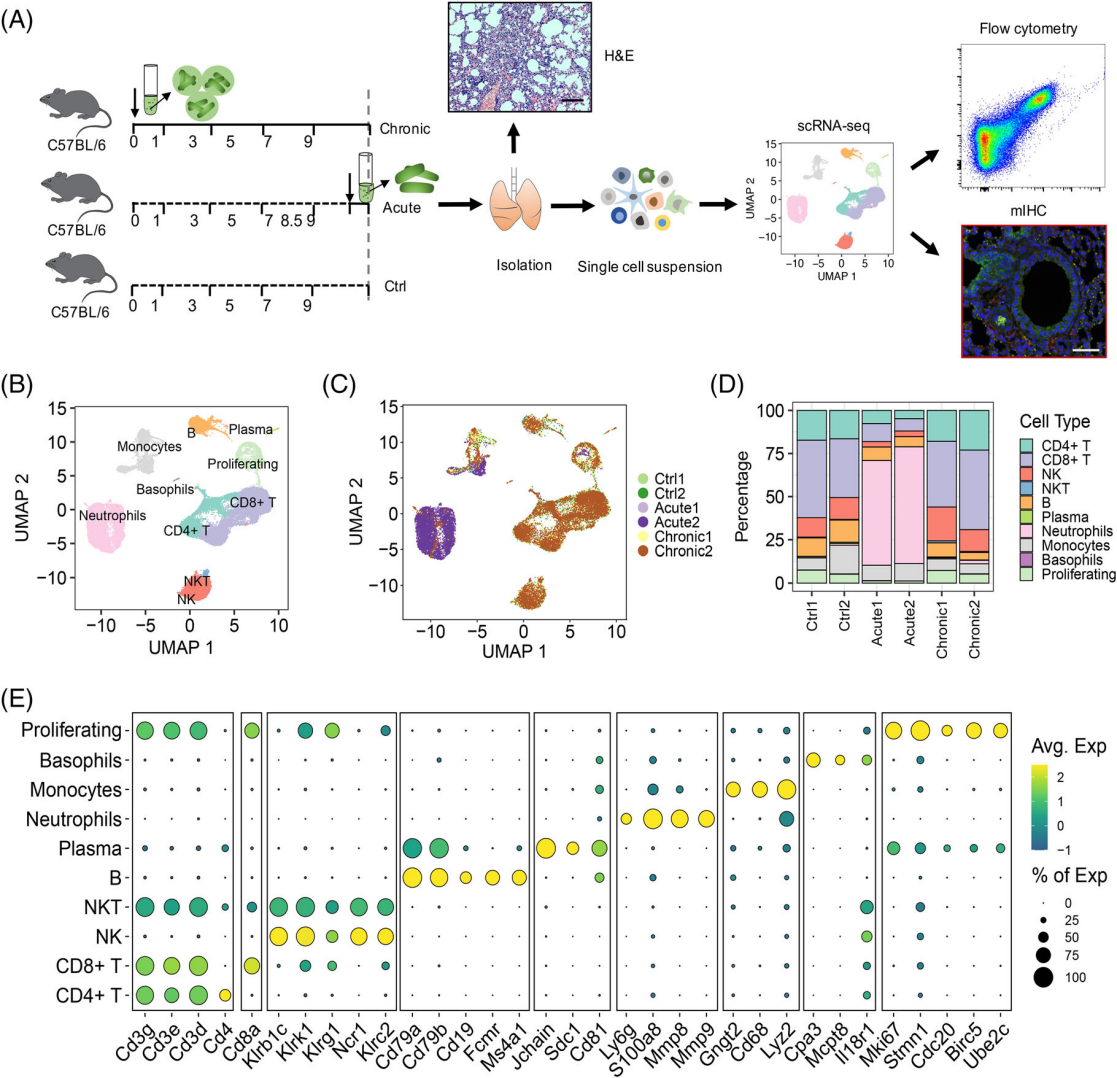
Single‐cell RNA sequencing (scRNA‐seq) profiling maps of immune cell gene expression in *Pseudomonas aeruginosa*‐infected lung tissues. (A) Wild‐type (WT) C57BL/6 female mice and those acutely and chronically infected with *P. aeruginosa* were isolated to generate a single‐cell suspension to perform scRNA‐seq (*n* = 2), which was validated by hematoxylin and eosin (H&E) staining (scale bar = 100 μm), fluorescent multiplex immunohistochemistry (mIHC) (scale bar = 100 μm), and flow cytometry. All figures are original (the mice, single‐cell suspension, and other elements were created by Adobe Illustrator). (B) Uniform manifold approximation and projection (UMAP) plot and graph visualization of all single cells defining 10 clusters. (C) UMAP plot and graph visualization of defining cell subclusters from six analyzed samples (Ctrl1, Ctrl2, Acute1, Acute2, Chronic1, Chronic2). (D) Bar plot showing the proportions of the 10 identified cell populations in each sample, colored according to cluster designation. Identified cell types are shown on the right. (E) Dotplot of the top expressed genes distinctly within the 10 clusters. Avg. Exp: average expression; NK, natural killer cells; NKT, natural killer T cells; % of Exp: percent of expression

To dissect the immune microenvironment of infected lung tissues, we established an acute infection model by transtracheal injection of planktonic *P. aeruginosa* reference strain PAO1 for 12 h and a well‐established and widely recognized chronic infection model by transtracheal injection of agar bead‐embedded PAO1 into C57BL/6 female mice for 10 days, similar to our previous report.^7^ Then, we collected lung tissues of healthy control (Ctrl), *P. aeruginosa* acute (Acute), and chronic (Chronic) infected mice with two biological replicates for each condition and dissociated these samples into a single‐cell suspension to carry out scRNA‐seq and bulk RNA‐seq (Figure 1A). Hematoxylin and eosin (H&E) staining was also applied to detect the injury degree of infected lung tissues, indicating more inflammatory cell infiltration after infection (Figure S1A). We then focused on CD45^+^ cells, enabling higher resolution analysis of the immune subsets infiltrated in the lung. We generated a single‐cell immune atlas with a total of 58,308 cells from healthy and infected mouse lung tissues with 10 clusters, including proliferating cells, CD4^+^ T cells, CD8^+^ T cells, B cells, plasma cells, natural killer (NK) cells, natural killer T (NKT) cells, neutrophils, monocytes, and basophils using well‐characterized cell marker genes (Figures 1B,E and S1B).

We further profiled the distribution of lung cells among different samples, showing considerable changes in the cell compositions (populations) during acute infection but relatively few alterations in multiple facets between healthy samples and chronically infected lungs (Figures 1C and S1C). Hierarchical clustering of cell abundances in each group revealed the proportion changes of clusters after acute and chronic pulmonary infection. Large amounts of neutrophils were recruited to the lungs during acute infection, while the proportions of other cell types were greatly reduced or had no significant changes in proportion. In addition, the proportions of T lymphocytes, NK cells, and B lymphocytes markedly increased, while monocytes were reduced in chronic infection samples (Figure 1D). Overall, these findings suggested an immune response involving infection‐induced expansion and changes in various immune cell subsets during bacterial infection in the lungs; importantly, there was a large contrast response between acute and chronic infection models.

### 2.2 Unique activation paths and distinct intercellular communications of T and NK cells contribute to dynamic immune responses

**FIGURE 2:**
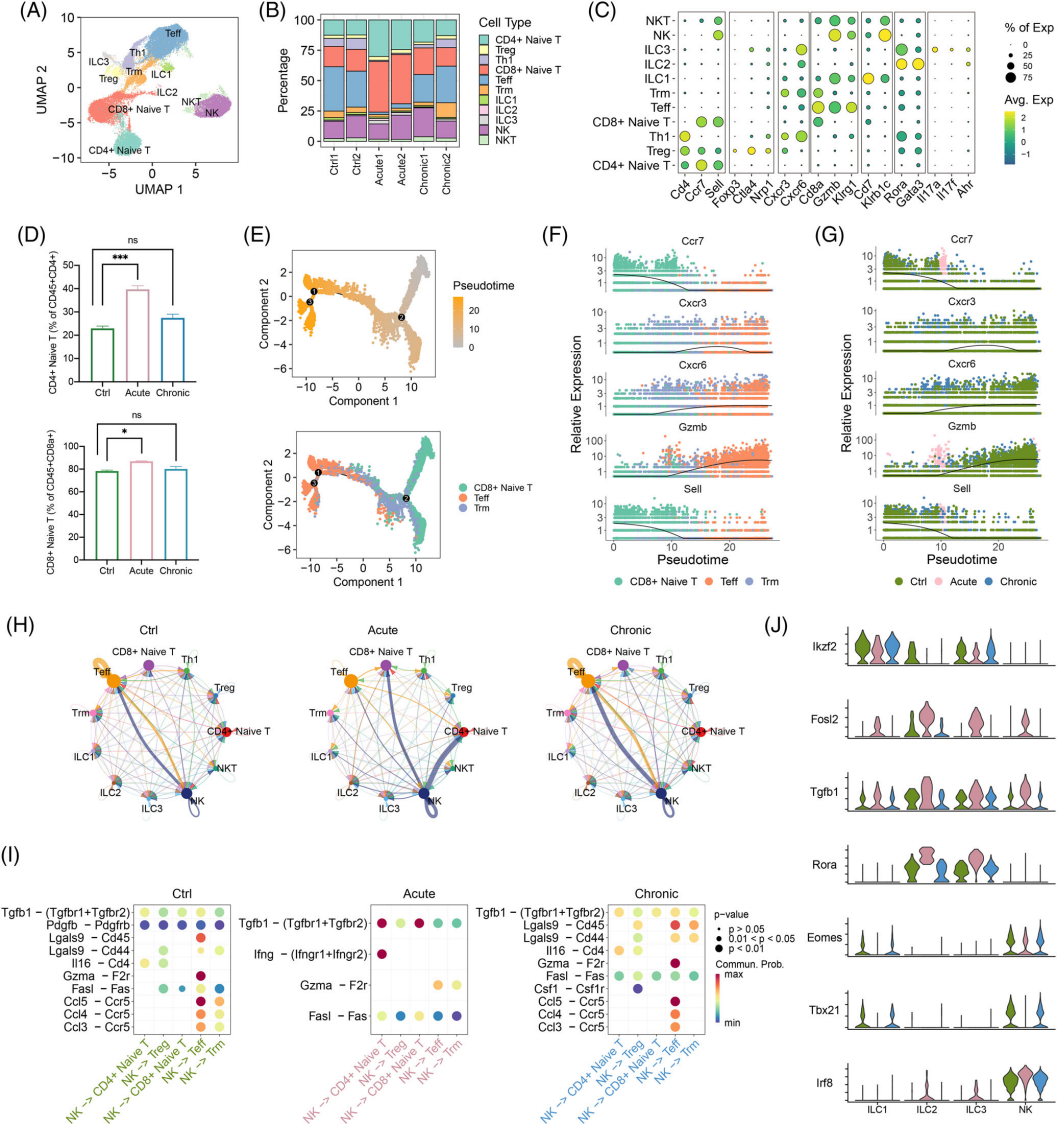
Dynamics of T and natural killer (NK) lymphocyte differentiation and cell–cell interaction under *Pseudomonas aeruginosa* infection. (A) Uniform manifold approximation and projection (UMAP) plot of the defined T‐ and NK‐cell subpopulations in the six samples. (B) Bar plot showing the proportions of the 11 identified cell populations in each sample, colored according to cluster designation. Identified cell types are shown on the right. (C) A dotplot of the top expressed genes distinctly within the 11 subclusters. Avg. Exp: average expression; % of Exp: percent of expression. (D) Histograms showing the proportion of CD4^+^ naïve T cells and CD8^+^ naïve T cells gated on CD45^+^CD4^+^CCR7^+^CD62L^+^ and CD45^+^CD8^+^CCR7^+^CD62L^+^, respectively (*n* = 3). Data are presented as the mean ± SEM, and representative data are shown of two separate experiments. Significant differences were designated by using analysis of variance (ANOVA) followed by Dunnett's multiple comparisons test. ns: *p* > 0.05, ^*^ *p* < 0.05, ^**^ *p* < 0.01, ^***^ *p* < 0.001, ^****^ *p* < 0.0001. (E) Pseudotime trajectories for CD8^+^ T cells (CD8^+^ naïve T, Teff, Trm) showed that different branches of CD8^+^ naïve T cells differentiated into Teff and Trm. (F) Profiling of marker genes along these trajectories to confirm their functional annotation: Ccr7, Sell, Cxcr3, Cxcr6, Gzmb for the linage of CD8^+^ T cells. (G) Density plots reflecting the marker genes along these trajectories stratified for the three groups: Ctrl, Acute, and Chronic. (H) Cell–cell interaction networks among T‐ and NK‐cell subsets estimated in the Ctrl, Acute, and Chronic groups based on CellChat. (I) Predicted interactions between NK and CD4^+^ naïve T, Treg, CD8^+^ naïve T, Teff, and Trm cells compared among the three groups. (J) Vlnplots of differentially expressed transcription factors (TFs) in NK, type 1 innate lymphoid cells (ILC1), type 2 innate lymphoid cells (ILC2), and type 3 innate lymphoid cells (ILC3) cells among the three groups. NKT, natural killer T cells; Teff, effector T cells; Th1, T helper type 1 cells; Treg, regulatory T cells; Trm, tissue resident memory T cells

To gain insights into T‐ and NK‐cell subpopulations, we extracted T lymphocytes and NK cells and performed further reclustering. We generated 11 clusters, including CD4^+^ naïve T cells, T helper type 1 cells (Th1), regulatory T cells (Treg), CD8^+^ naïve T cells, effector T cells (Teff), tissue resident memory T cells (Trm), type 1, 2, 3 innate lymphoid cells (ILC1, ILC2, ILC3), NK cells, and NKT cells. The definitions of these cell subsets referred to the databases (Mouse Cell Atlas, CellMarker and PanglaoDB) and previous reports^3,^ ^17^, ^18^ (Figures 2A–C and S2A,B). The ratio analysis of lymphocytes in the lung samples showed large abundance changes in these cell subsets. In detail, CD4^+^ and CD8^+^ naïve T cells increased considerably after acute infection, indicating activation of the adaptive immune response in the early stage at 12 h. The majority of subpopulations showed no significant changes in chronic infection samples, with only Trm cells increasing to a certain extent, indicating that the process of chronic infection exhibits a constant state (Figure 2C). In addition, we performed flow cytometry to validate the proportion changes of CD4^+^ naïve T cells, CD8^+^ naïve T cells, and Teff cells among the three groups, which were consistent with the sequencing results (Figure 2D).

Next, we assessed the gene differentiation of CD8^+^ and CD4^+^ T cells and focused on the changes in several gene signatures in these subsets by applying a monocle R package to analyze their pseudotime trajectories. Specifically, CD8^+^ T‐cell subsets differentiated from naïve to effector and memory states, and their marker genes (Ccr7, Sell, Cxcr3, Cxcr6, and Gzmb) were consistent with the state changes (Figure 2E,F). Furthermore, density plots reflecting the relative number of cell subsets in each phenotypic state were created along with these trajectories, which showed that CD8^+^ T cells in the acute infection group were enriched in the CD8^+^ naïve T lineage, while the Teff and Trm lineages were enriched in the Ctrl and Chronic groups (Figure 2G). Similarly, the CD4^+^ T‐cell subsets also showed consistent dynamics of the pseudotime trajectory with CD8^+^ T‐cell subsets (Figure S3A–C). We also used gene set enrichment analysis (GSEA) to analyze the differentially expressed genes of CD4^+^ and CD8^+^ naïve cells between the Acute and Ctrl groups. Genes (Socs3, Stat3, Jak2, Nfkbia, and Mapk9) related to Janus kinase/signal transducer and activator of transcription (JAK/STAT), the RIG‐I receptor signaling pathway, and the MAPK signaling pathway were enriched in the acute infection group, indicating that CD4^+^ and CD8^+^ naïve T cells respond intensively and upregulate genes to activate the immune response to cope with invasion challenges in acute infection (Figure S3D,E).

Recent evidence has shown that, in addition to cell composition, the interactions of immune cells can also affect immune response outcome. Thus, we performed cell–cell interaction analysis among the three groups using the CellChat package to further investigate the dynamics of cell–cell communication upon *P. aeruginosa* infection. In general, the cell–cell interactions of cell subtypes changed considerably in the Acute and Chronic groups compared with the Ctrl group, which changes mainly occurring in the interactions among NK cells, Teff cells, CD4^+^ naïve T cells, CD8^+^ naïve T cells, Tregs, and Trm cells. Specifically, in the Acute group, NK cells mainly interacted with naïve CD4^+^ and naïve CD8^+^ T cells. However, the interactions between NK cells and Teff cells were dominant in the Ctrl and Chronic groups. Significantly, the interaction strengths of NK cells and Teff were stronger in the Chronic group than in the other two groups (Figure 2H). Furthermore, ligand–receptor pairs involved in phenotypic interaction changes also exhibited significant differences among the three groups. Compared to the Ctrl group, two ligand–receptor pairs encompassing Tgfb1‐Tgfbr1/Tgfbr2 and Ifng‐Ifngr1/Ifngr2 in the interactions of NK cells with CD4^+^ naïve and CD8^+^ naïve T cells increased in the Acute group, while Tgfb1‐Tgfbr1/Tgfbr2, Lgals9‐CD45, and Lgals9‐CD44 in the interactions of NK cells with Teff and Trm cells increased in the Chronic group (Figure 2I). In general, these results showed highly dynamic changes in cell–cell interactions and ligand–receptor pairs among the three groups in T‐ and NK‐cell subtypes.

Next, we profiled transcription factors (TFs) of T‐ and NK‐cell subtypes, showing that several TFs were highly expressed in each cluster (Figure S3F). We found that among the three groups, Ikzf2, a member of the Ikaros TF family that regulates lymphocyte development and maintains the function of T cells,^19^, ^20^ was expressed at lower levels in the Acute and Chronic groups than in the Ctrl group, mainly in ILCs (ILC1, ILC2, ILC3). In contrast, several TFs related to the regulation of ILC development,^21^ airway inflammation,^22^ and alveolar regeneration,^23^ including Fosl2, Tgfb1, and Rora, increased considerably in the Acute group. Furthermore, Eomes, critical for NK‐cell development and cytotoxic function,^24^ was downregulated in the Acute group, whereas Irf8, an important regulator of virus‐specific NK cells,^25^ was upregulated, suggesting that NK cells attempt to maintain balanced TF regulation when facing acute *P. aeruginosa* infection. Additionally, Tbx21 (encoded T‐bet), regulator of lymphocyte differentiation,^26^ was downregulated in the Chronic group, indicating that Tbx21 may mediate the functional dysregulation of ILC1s and NK cells in chronically infected conditions (Figure 2J). Therefore, these findings suggested that during infection, many TFs of ILCs regulated the process actively or inactively in response to *P. aeruginosa* infection.

### 2.3 Cxcl2^+^ B cells are expanded and activated in acute pulmonary infections

**FIGURE 3:**
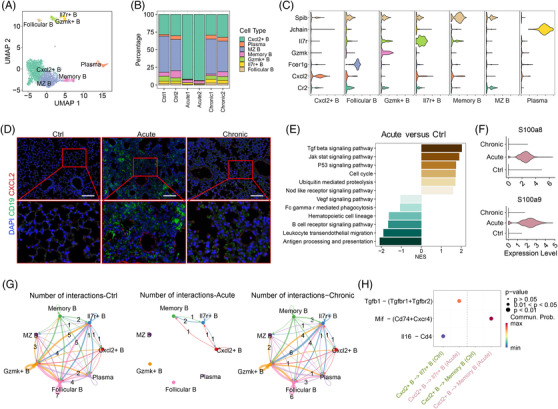
Cxcl2^+^ B cells are strongly enriched in acute pulmonary infection. (A) Uniform manifold approximation and projection (UMAP) plot of the defined B‐cell subsets in the six samples. (B) Bar plot showing the relative proportions of the seven identified cell populations in each sample, colored according to cluster designation. Identified cell types are shown on the right. (C) Vlnplot of the marker genes distinctly expressed within the seven subclusters. (D) Multiplex immunohistochemistry (mIHC) showing coexpression of CD19 (green), Cxcl2 (red), and 4′,6‐diamidino‐2‐phenylindole (DAPI) (blue)‐positive cells called Cxcl2^+^ B cells among the three groups. Magnification: 20×. Scale bar = 100 μm. (E) Gene set enrichment analysis (GSEA) of differentially expressed genes between the Acute and Ctrl groups in Cxcl2^+^ B cells, showing several pathways enriched in the Acute and Ctrl groups. (F) Vlnplots of S100a8 and S100a9 differentially expressed in Cxcl2^+^ B cells among the three groups. (G) Networks of cell–cell interactions in B‐cell subsets among the three groups. (H) Predicted interactions between Cxcl2^+^ B cells and Il7r^+^ B and memory B cells, comparing Ctrl and Acute. MZ B, marginal zone B cell

B cells are an important bulwark of acquired immunity, and the presence of B cells in inflammatory infiltrates is correlated with disease severity in many pulmonary diseases.^27^, ^28^ Here, we identified seven B‐cell subpopulations, Cxcl2^+^ B cells, follicular B cells, Gzmk^+^ B cells, Il7r^+^ B cells, memory B cells, marginal zone B cells, and plasma B cells, according to their distinct markers (Figures 3A–C and S4A). Specifically, Cxcl2^+^ B cells were highly increased and distributed across almost all B‐cell subsets in acute infection (Figures 3B and S4B). We next applied multiplex immunohistochemistry (mIHC) to validate this large change among the three groups and found that Cxcl2 increased significantly in Cxcl2^+^ B cells expressing CD19 in the Acute group but not in the Chronic group (Figure 3D). We analyzed the differentially expressed genes of Cxcl2^+^ B cells between the Ctrl and Acute groups (Figure S4C) and further performed GSEA of these genes, which found that the transforming growth factor‐beta (TGF‐β), JAK/STAT, and nucleotide binding oligomerization domain (NOD)‐like signaling pathways were upregulated in the Acute group (Figure 3E). Moreover, Cxcl2^+^ B cells expressed much higher levels of S100a8 and S100a9 after acute infection (Figures 3F and S4C). The chemokine Cxcl2 can recruit neutrophils to infected sites, further causing the activation of inflammation.^29^ S100A8/9 are calcium‐binding proteins and can be secreted into the extracellular space to activate immune responses.^30^ Therefore, S100a8/9 and Cxcl2 may help induce the inflammatory response in acute infection, indicating that they play a positively regulated role in acute infection.

Given the potential important role of Cxcl2^+^ B cells, we investigated the interactions between Cxcl2^+^ B cells and other B‐cell subsets. The analysis results revealed large changes in the Acute group compared to others, and only Cxcl2^+^ B cells, Il7r^+^ B cells, and memory B cells interacted with each other, indicating that cell–cell interactions became centralized in the Acute group (Figures 3G and S4D). Further analysis of ligand–receptor pairs in the Acute group showed that the expression levels of TGF‐β (Tgfb1) and its multimeric Tgfbr1 and Tgfbr2 receptors increased when Cxcl2^+^ B cells interacted with Il7r^+^ B cells, while the levels of macrophage migration inhibitory factor (Mif) and its multimeric Cd74 and Cxcr4 receptors increased when Cxcl2^+^ B cells interacted with memory B cells (Figure 3H). Above all, these findings suggested that Cxcl2^+^ B cells increased in proportion and cell–cell interactions with other cell subsets after acute infection.

### 2.4 Diverse functional changes in myeloid cells in infected lung lesions

**FIGURE 4:**
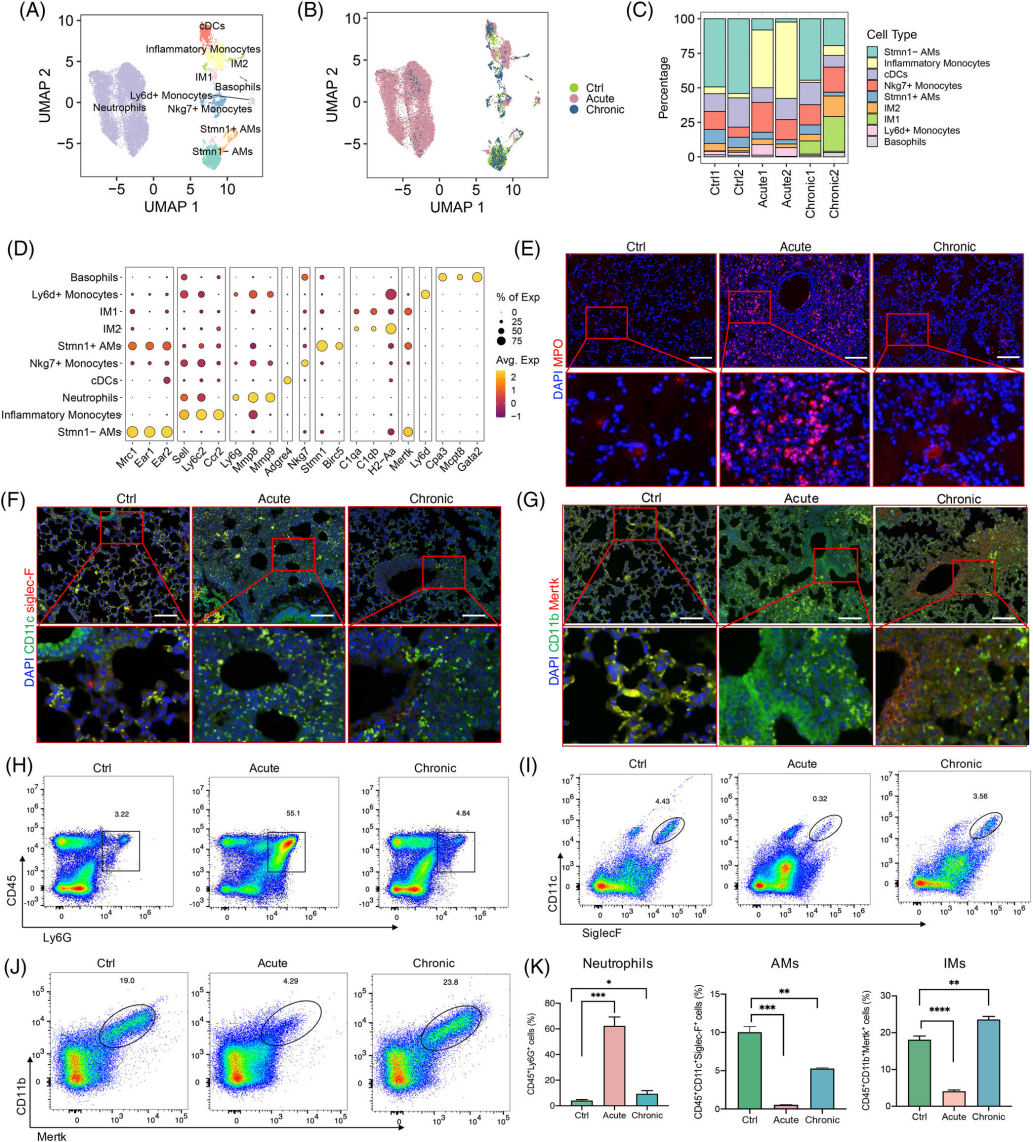
Deciphering the diversity of myeloid cells in infected lung lesions. (A) Uniform manifold approximation and projection (UMAP) plot of the defined myeloid cell subsets in the six samples. (B) UMAP plot of the myeloid cell subsets in the three groups. (C) Bar plot showing the relative proportions of identified cell populations in each sample, colored according to cluster designation. Identified cell types are shown on the right. (D) Dotplot of the top expressed genes distinctly within the subclusters of myeloid cells. Avg. Exp: average expression; % of Exp: percent of expression. (E) Immunofluorescence (IF) of neutrophils with 4′,6‐diamidino‐2‐phenylindole (DAPI) (blue) and myeloperoxidase (MPO) (red) colored among the three groups is shown. Magnification: 20×. Scale bar = 100 μm. (F and G) Multiplex immunohistochemistry (mIHC) showing coexpression of CD11c (green), Siglec‐F (red), and DAPI (blue)‐positive cells called alveolar macrophages (AMs) (F) and CD11b (green), Mertk (red), and DAPI (blue)‐positive cells called interstitial macrophages (IMs) (G) among the three groups. Magnification: 20×. Scale bar = 100 μm. (H–J) Representative flow cytometry plots and quantification showing the proportional changes in neutrophils (H), AMs (I), and IMs (J) in the three groups. (K) Histograms showing the proportion of neutrophils, AMs and IMs among the three groups (*n* = 3). Bars represent mean ± SD. Significant differences were designated by using analysis of variance (ANOVA) followed by Dunnett's multiple comparisons test. ^*^ *p* < 0.05, ^**^ *p* < 0.01, ^***^ *p* < 0.001. All data are shown representative of two separate experiments. cDCs, conventional dendritic cells; IM1, type 1 interstitial macrophages; IM2, type 2 interstitial macrophages; Stmn1^+^ AMs, Stmn1^+^ alveolar macrophages; Stmn1^−^ AMs, Stmn1^–^ alveolar macrophages

During severe bacterial infection, regeneration of myeloid cells is highly active due to the unique pattern in combating invading pathogens.^31^, ^32^ To understand the dynamics of myeloid cells in pulmonary infection at the single‐cell level, we identified nine distinct subsets after dimensional reduction, including neutrophils, basophils, Stmn1^+^ alveolar macrophages (Stmn1^+^ AMs), Stmn1^−^ alveolar macrophages (Stmn1^−^ AMs), type 1 interstitial macrophages (IM1), type 2 interstitial macrophages (IM2), conventional dendritic cells, inflammatory monocytes, Ly6d^+^ monocytes, and Nkg7^+^ monocytes, according to their typical marker genes (Figures 4A–D and S5A).

We further analyzed the distribution changes of these subsets among the grouped samples. A large expansion of neutrophils in acute infection samples (Figure 4B) indicated that neutrophils responded to acute infection and were quickly recruited from peripheral tissues to infectious sites. In contrast, Stmn1^+^ AM and Stmn1^−^ AM were decreased in the Acute and Chronic groups compared to the Ctrl group (Figure 4C). However, strong expansion of IM1 was notably expanded in the Chronic group, rather than the Acute group, which may be a potential cell biomarker of chronic pulmonary infection. In addition, Nkg7^+^ monocytes, Ly6d^+^ monocytes, and inflammatory monocytes increased in acute infection samples, which likely underwent activation with the onset of acute infection (Figure 4C). Moreover, according to their altered proportions, we confirmed the significant changes in neutrophils, AMs and IMs among the three groups using mIHC and flow cytometry analyses (gating strategies are shown in Figure S5B), which is consistent with the scRNA‐seq analysis results (Figure 4E–K). In general, these findings suggested that with the exception of neutrophils responding to infections initially and quickly, other myeloid subsets, such as IMs, AMs, and inflammatory monocytes, were also activated and regenerated constantly to defend against *P. aeruginosa* invasion in both Acute and Chronic models. Further analysis and illumination of these subclusters in gene signatures and dynamic changes in cell–cell interactions will be intriguing.

**FIGURE 5:**
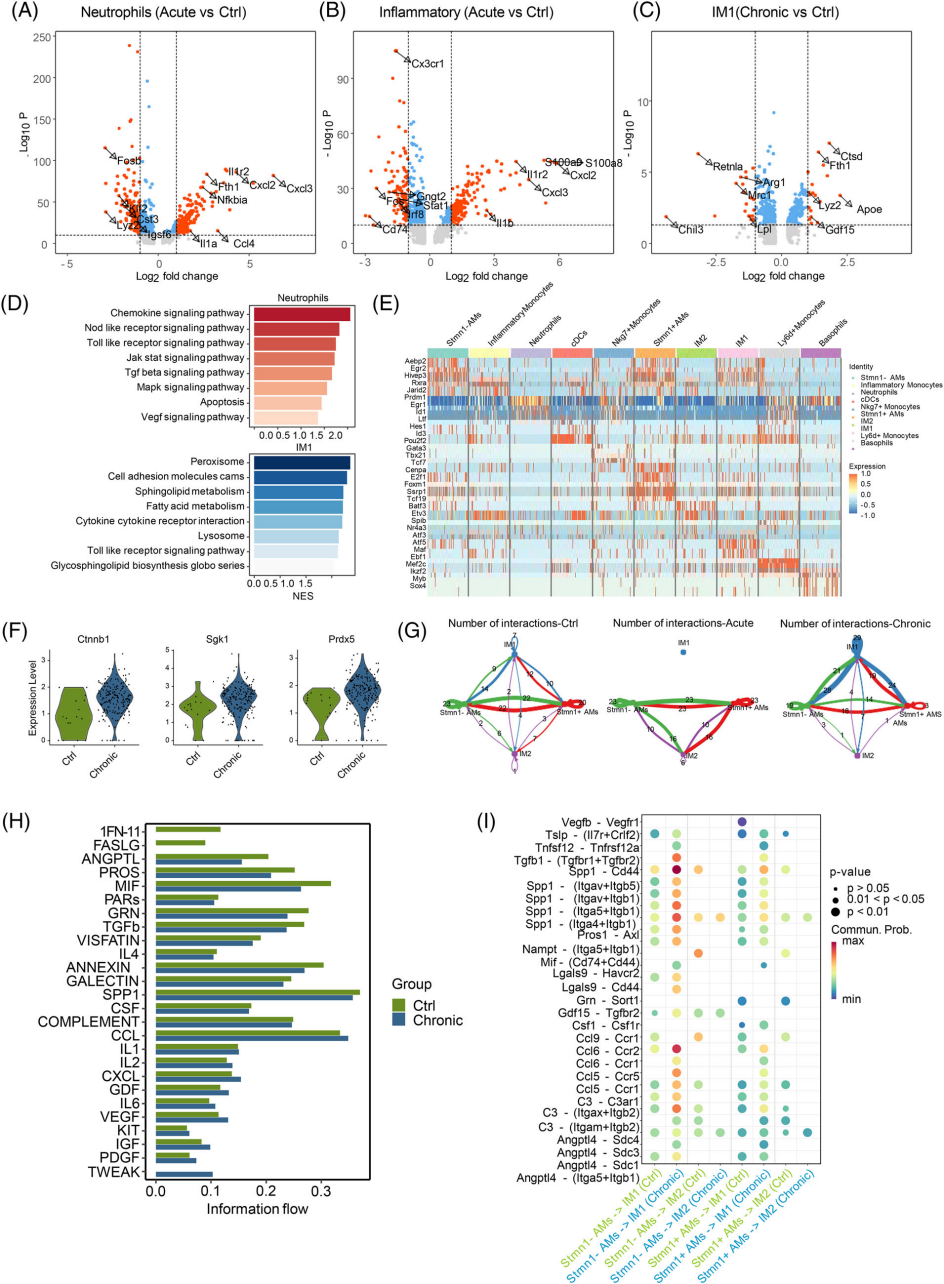
Neutrophils, interstitial macrophages (IMs), and alveolar macrophages (AMs) have distinct signatures upon pulmonary infection. (A–C) Volcano plots of the differentially modulated genes of neutrophils in the Acute versus Ctrl group (A), inflammatory monocytes in the Acute versus Ctrl group (B), and type 1 interstitial macrophages (IM1) in the Chronic versus Ctrl group (C). The *x*‐axis specifies the average log2‐fold change (FC), and the *y*‐axis specifies the negative logarithm (base 10) of the adjusted *p*‐values. Left of the threshold value dots indicate genes whose expression is downregulated, right dots indicate genes whose expression is upregulated. Representative genes are labeled. (D) Gene set enrichment analysis (GSEA) of highly expressed genes of neutrophils and IM1 showed several signaling pathways increased in the Acute and Chronic groups, respectively (*p* < 0.05, NES > 1). (E) Heatmap of differentially expressed transcription factors (TFs) of myeloid cell subsets. (F) Vlnplots of TFs, including Ctnnb1 Sgk1 and Prdx5, in IM1 differentially expressed between the Ctrl and Chronic groups. (G) Number of cell–cell interactions of IM1, type 2 interstitial macrophages (IM2), Stmn1^+^ alveolar macrophages (Stmn1^+^ AMs), and Stmn1^–^ alveolar macrophages (Stmn1^−^ AMs) among the three groups. (H) Overview of the signaling pathway related to (F) comparing the Ctrl and Chronic groups. (I) Predictive interactions in IM1, IM2, Stmn1^+^ AMs, and Stmn1^−^ AMs between the Ctrl and Chronic groups

Given that the distribution of myeloid cell subtypes changed considerably upon infection, we first focused on how differential genes and TFs affected these significantly changed signatures. Neutrophils, the well‐known first‐line immune cells recruited to the site of inflammation, increased considerably after acute pulmonary infection. Similarly, inflammatory monocytes also expanded markedly in the Acute group. Differential gene analysis of neutrophils and inflammatory monocytes showed that compared to the Ctrl group, the Acute group expressed high levels of proinflammatory genes and chemokines, such as Il1r2, Cxcl2, and Tlr2, which were enriched in chemokines, Toll‐like receptors, NOD‐like receptors, and other signaling pathways related to activation of the immune response (Figure 5A,B,D). Bulk RNA‐seq analysis also showed consistent results in the Acute group in gene signatures, biological processes, and enriched signaling pathways (Figure S6A–C). Therefore, these results indicated that the myeloid cell subsets showed large proportions and related signaling pathway changes in response to infections, whether acutely or chronically.

### 2.5 IMs and AMs have distinct functions in shaping immune systems during pulmonary infections

A previous study showed that AMs include a type of subset called M2‐type population which is suitable for Mycobacterium *tuberculosis* replication,^8^, ^33^ while environment of IMs is more stressful for the bacteria.^8^, ^34^ Hence, we focused on their changes in gene signatures, related signaling pathways and biological processes. First, we found that the two subtypes of AMs exhibited clearly different gene signatures and enriched signaling pathways. In particular, Stmn1^+^ AMs expressed higher levels of proliferative genes, such as Top2a (DNA Topoisomerase II Alpha) and Ccl2 (C‐C Motif Chemokine Ligand 2), while Stmn1^−^ AMs expressed higher levels of Mrc1 (encoding CD206, marker gene of M2 macrophages) and Slc7a2 (solute carrier family 7 member 2, important for macrophage function) (Figure S7A). We focused on the differentially expressed genes and enriched signaling pathways of these two cell types themselves. We found that these differentially expressed genes were enriched in chemokines and NOD‐like receptor signaling pathways in Stmn1^+^ AMs, while proteasome and Peroxisome proliferator‐activated receptor (PPAR) signaling pathways were enriched in Stmn1^−^ AMs (Figure S7B), suggesting that these two subtypes might play different roles in the progression of infection. Stmn1^+^ AMs proliferate and secrete chemokines to recruit other cells to clear pathogens, while Stmn1^−^ AMs prefer to play a protective and constant role in phagocytosing pathogens. We also took note of a TF and a marker gene of M2 macrophages, Egr2, which can bridge the early transient and late stable gene expression program of polarization.^35^ Egr2 was highly expressed in AMs from healthy lung tissues but significantly downregulated during chronic infection, indicating that the polarization phenotype of AMs was weakened to a certain extent (Figures 5E and S7C).

Next, we investigated the dynamics of IMs that dominated in the Chronic group. Gene differential analysis of IM1, which was increased only in chronic infection and not in acute infection, expressed high levels of cathepsin D (Ctsd), apolipoprotein E (ApoE), and lysozyme 2 (Lyz2), and these genes were enriched in the lysosome, peroxisome, and Toll‐like receptor signaling pathways, implying that IM1 expanded after chronic infection to activate and mediate the elimination of pathogens (Figure 5C,D). Notably, ApoE was expressed at higher levels in IM1 in the Chronic group than in the Ctrl group, indicating that it plays an antibacterial role during chronic infection (Figure 5C). This finding is consistent with a previous study showing that ApoE possessed antibacterial activity like *P. aeruginosa* and *Escherichia coli*.^36^ Moreover, it was reported that Fth1 (encoding ferritin heavy chain 1) could inhibit ferroptosis and that Fth1‐deficient bone marrow‐derived macrophages exhibited marked increases in susceptibility to immune stimulation and oxidative stress.^37^, ^38^ Here, Fth1 was upregulated in chronic infection, suggesting that Fth1 could protect IM1 from ferroptosis (Figure 5C). These findings were consistent with the results of bulk RNA‐seq, demonstrating that ApoE and Lyz2 and their related signaling pathways were increased in the Chronic group (Figure S6A,B,D). We also analyzed the transcription profiles of IM1 between the Ctrl and Chronic groups. The results presented increased expression of several TFs, including catenin beta 1 (Ctnnb1), serum/glucocorticoid regulated kinase 1 (Sgk1), and peroxiredoxin 5 (Prdx5), indicating that these TFs may be involved in the regulation of chronic *P. aeruginosa* infection (Figures 5F and S7C). Our results were consistent with a previous report that activation of the β‐catenin‐SGK1‐Foxo axis was related to the function of interferon (IFN)‐γ^+^ Treg cells and autoimmunity,^39^ indicating that this observation may have broad relevance. To further verify the function of IMs in the control and chronic infection groups, we depleted IMs by the CSF1r inhibitor PLX5622 without any effect on AMs according to the previous report.^40^ IMs were sorted by flow and adopted into the lungs of control and chronically infected mice via intrabronchial administration (Figure S8A). We also detected the IMs after chowed PLX5622 for 3 days to verify the depletion of IMs (Figure S8B). Our results showed that the mice with adoption of IMs exhibited higher survival rate and lower bacterial load (Figure S8C,D). In addition, H&E staining showed some damage repair and less inflammatory infiltration of the lung tissues in mice with adoption of IMs (Figure S8E). Besides, detection of CD4^+^ and CD8^+^ T cells by flow cytometry indicated that the mice with reinfusion of IMs occupied enhanced adapted immune response (Figure S8F,G). Thus, IMs appears to have a protective role for host likely by repressing the progress of chronic bacterial infection.

Given the important function of macrophages and the above significant changes in AMs and IMs in the process of infection, we further analyzed cell–cell interactions of myeloid cell subsets, especially the four macrophage subsets among the three groups (Figure S9A). As expected, the number of interactions changed considerably among the three groups. In detail, only IM2, Stmn1^+^ AMs, and Stmn1^−^ AMs interacted with each other in the Acute group, while all four cell clusters interacted with each other in the other two groups. Notably, an increase in the interaction between IM1 and other subsets occurred in the Chronic group (Figure 5G). We then compared the overall signaling pathways in macrophage subsets between the Ctrl and Chronic groups and found that the signaling pathways involved in chemokine and proinflammatory cytokine expression were significantly different. CC chemokine ligand (CCL), C‐X‐C motif chemokine ligand (CXCL), and interleukin 1 (IL‐1) were expressed at higher levels in the Chronic group than in the Ctrl group (Figures 5H and S9B). In addition, we found that IM1 can also interact with lymphocyte subsets, such as NK, NKT, Treg, Th1, Trm, and Teff cells, mainly through chemokines and their receptors, including Ccl5, Ccl6, Cxcl4, and Cxcl16 (Figure S9C). Further ligand–receptor analysis revealed that in chronic infection, Spp1, Ccl5, Ccl6, and Tgfb1 with their receptors were upregulated through the interaction of Stmn1^−^ AMs and IM1, emphasizing that this interaction may help recruit other immune cells to eliminate pathogens and subsequent tissue repair (Figures 5I and S9D). In summary, IMs and AMs showed different responses in proportion, gene expression, and cell–cell interactions to acute and chronic pulmonary infections, respectively.

### 2.6 Healthy and diseased mouse lungs display high variation in cell–cell interactions

**FIGURE 6:**
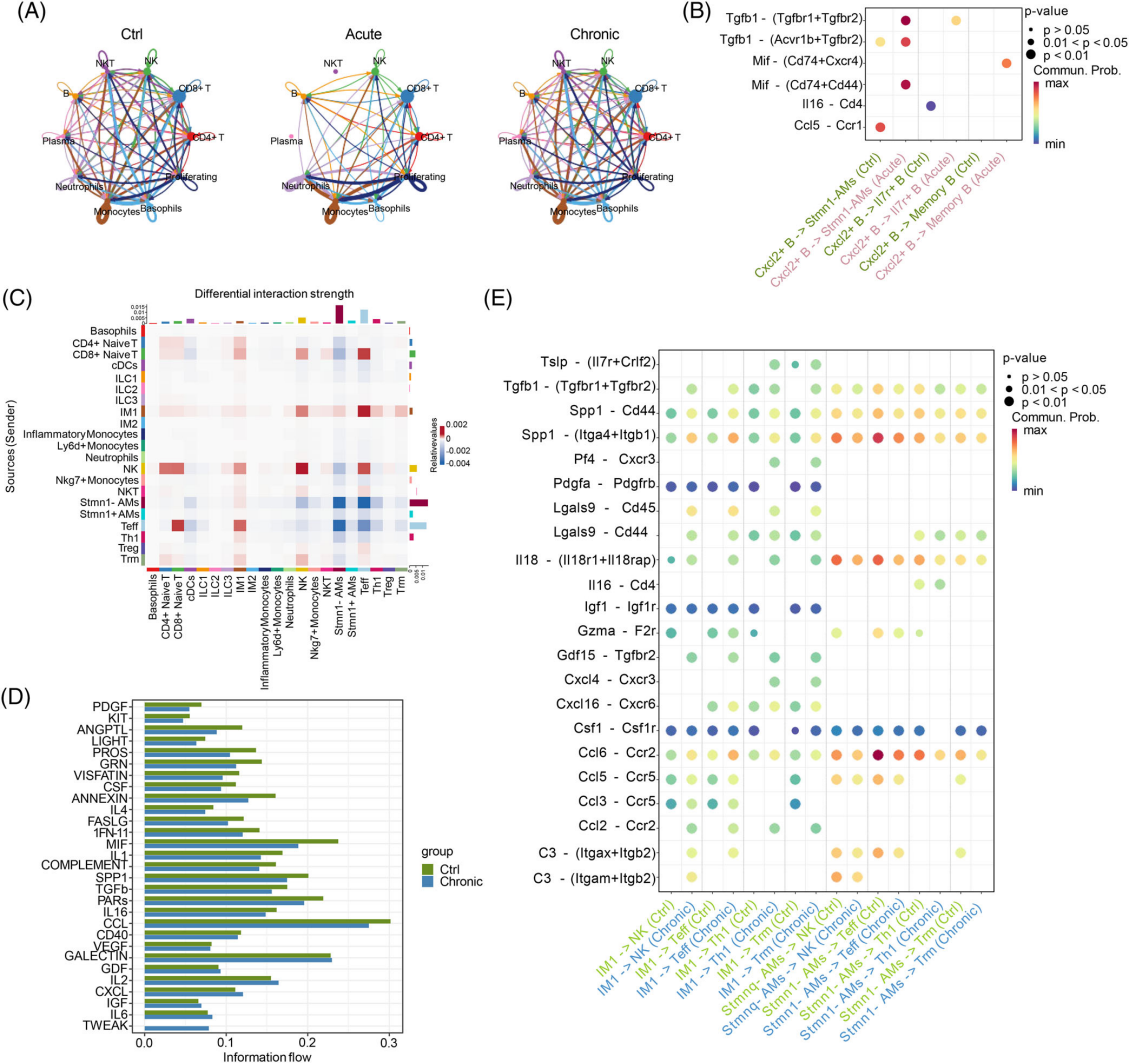
Healthy and diseased mouse lung tissues display high variation in cell–cell interaction. (A) Number of predicted interactions (*p* ≤ 0.05) between immune cell subsets based on CellChat in the three groups. (B) Predictive interactions of Cxcl2^+^ B cells, Stmn1^–^ alveolar macrophages (Stmn1^−^ AMs), Il7r^+^ B cells, and memory B cells between the Ctrl and Acute groups. (C) Differences in the strength of interactions between myeloid, T and natural killer (NK) lymphocyte subsets, comparing Chronic versus Ctrl, showing generally stronger interactions in Chronic group. (D) Overview signaling pathway comparison related to interactions of myeloid, T and NK cell subsets in the Ctrl versus Chronic groups. (E) Predictive interactions of type 1 interstitial macrophages (IM1), NK, Stmn1^−^ AMs, effector T cells (Teff), T helper type 1 cells (Th1), and tissue resident memory T cells (Trm) between the Ctrl and Chronic groups. ILC1, type 1 innate lymphoid cells; ILC2, type 2 innate lymphoid cells; ILC3, type 3 innate lymphoid cells; IM2, type 2 interstitial macrophages; NKT, natural killer T cells; Stmn1^+^ AMs, Stmn1^+^ alveolar macrophages; Treg, regulatory T cells

Since our data identified that large amounts of neutrophils were recruited in acute infection, yet T‐cell lineages were disrupted, so we further explored the interactome among different cell types to gain deeper insights. First, we calculated interactions of all cell types separately for the three groups, and then we analyzed the number and strength of specific interactions. The numbers of proliferating cells and neutrophils interacting with other immune cells in the Acute group were greater than those in the other two groups. Conversely, specific interactions were predicted among immune cells, especially in the Ctrl and Chronic groups (Figure 6A). Furthermore, the interaction strength of the cell–cell interaction was stronger between neutrophils, monocytes, and proliferating cells in the Acute group than in the Ctrl group, and stronger interactions between CD4^+^ T, CD8^+^ T, NK, B, plasma, neutrophils, and proliferating cells were observed in the Chronic group (Figure S10A,B), suggesting that cellular activity in acute infection is relatively less broad but more intensive than that in chronic infection.

We then investigated the interaction details between myeloid and lymphocyte subpopulations. The results showed that communication between Cxcl2^+^ B cells and Stmn1^−^ AMs became stronger via Tgfb1‐Tgfbr1/Tgfbr2, Tgfb1‐Acvr1b/Tgfbr2, and Mif‐Cd74/Cd44 after acute infection (Figure 6B). We noticed that major cell subtypes (NK cells, CD4^+^ naïve T cells, CD8^+^ naïve T cells, Teff cells, and IM1 cells) interacted with each other more strongly in the Chronic group than in the Ctrl group (Figure 6C), indicating that during chronic infection, the dynamics of cell–cell communication became stronger in response to *P. aeruginosa* challenge. Given the strong dynamic changes in cell–cell interactions in the process of infection, we next analyzed their effects on integral signaling pathways among the three groups and observed that among the chemokines and interferon signaling pathways, the CCL cluster, IL‐6, the CXCL cluster, type I interferon (IFN‐I), and tumor necrosis factor were activated after acute infection (Figure S10C). Similarly, IL‐6‐, IL‐2‐, and CXCL‐related signaling pathways were also activated in chronic infection (Figures 6D and S10D). Furthermore, ligand–receptor analysis showed that in acute infection, the main interaction between neutrophils and proliferating cells occurred through high expression levels of chemokine and chemokine receptors to recruit other types of cells to the infected sites, which may be required for host defense (Figure S10E). However, the cell–cell interactions and specific ligand–receptor pairs exhibited a more complex status in chronic infection. In detail, the myeloid cell subtypes were regulated to the lymphocyte subtypes mainly through higher ligand–receptor pairs of secreted phosphoprotein 1 (Spp1) with its multimeric Itga4 and Itgb1 receptors in the Chronic group and through lower ligand–receptor pairs of chemokine–chemokine receptors, including Ccl2, Ccl3, and Ccl5 (Figures 6E and S10F). In general, these results showed overall changes in cell–cell interactions in response to different infection conditions, which revealed many ligand–receptor pairs related to the inflammatory response during infectious processes.

## 3 DISCUSSION

*P. aeruginosa* is an opportunistic pathogen that often causes serious acute and chronic infections clinically, especially in immunocompromised people, and the interaction of *P. aeruginosa* with the host needs to be deeply elucidated to provide effective therapeutic strategies. Our efforts to understand the mechanisms of bacterial infections, especially chronic infections, have constantly been stymied by the heterogeneity of immune cells within *P. aeruginosa*‐infected tissues. In this study, we applied scRNA‐seq to generate a profiling of 58,308 immune cells including cellular composition, distribution, and functional activity in healthy and *P. aeruginosa*‐infected mouse lung tissues.

By decrypting the immune cell atlas of *P. aeruginosa*‐infected lungs under both acute and chronic conditions, we identified 10 cell clusters. We captured different changes in immune cell subpopulations during healthy and infectious conditions. Importantly, *P. aeruginosa* infection affected the composition, distribution, and expression patterns of cellular compartments necessary for host immune defense. Within T and B lymphocyte clusters, we found that CD4^+^ naïve T, CD8^+^ naïve T, and Cxcl2^+^ B cells expanded markedly after acute infection, and further enrichment analysis indicated their positive regulation of the immune response. Meanwhile, inflammatory monocytes and neutrophils also increased significantly in response to acute infection. The findings are partly consistent with human studies. Specifically, the neutrophils expanded a lot after the acute infection and also increased after chronic infection, which totally accords with previous reports.^41^, ^42^ Remarkably, the proportion of IM cells showed a considerable increase, while that of AM cells decreased significantly in the Chronic group. A recent study applied scRNA‐seq to detect the sputum of CF patients and revealed that the neutrophils were the most prevalent, while the AMs were reduced a lot in CF, which were partly consistent with our findings of Chronic group.^43^ Furthermore, we found that the two subtypes of AMs played different roles in host defense against chronic *P. aeruginosa* infection. Within IMs, they expressed high levels of genes related to macrophage activation and antibacterial activity, indicating that IMs play an important role in helping the host clear pathogens when facing chronic infection. Critically, the cluster annotations of the above cells were largely consistent with those in previously published datasets.^3^, ^44^, ^45^ Thus, our data provide further indications that Cxcl2^+^ B cells and IMs may strongly connect to *P. aeruginosa* pathophysiology and have great potential for designing effective therapeutic strategies.

Our study also highlights the importance of the highly dynamic cell–cell interaction process in different pathological conditions. Here, we provide a data‐driven transcriptomic atlas identifying immune cell subpopulations with the greatest alterations and the molecular pathways activated in response to *P. aeruginosa*. In particular, our data characterized critical macrophages and neutrophils as important immune cells participating in dynamic cellular crosstalk during *P. aeruginosa* infection. Different immune cell subsets interacted with others through various ligand–receptor pairs mainly involving chemokine–chemokine receptors and Spp1 with its receptors, which dominated the interaction between macrophage and lymphocyte subsets. Specifically, the chemokine–chemokine receptors showed higher dynamics in the Acute group, while Spp1 with its receptors exhibited higher dynamics in the Chronic group. These findings suggested that host immune cells enhanced cell–cell interaction dynamics through various ligand–receptor pairs to combat pathogens upon acute or chronic infections.

We note several limitations to this study. First, gender is a significant biological factor that affects the functions of the immune system, while only female mice were used in our study.^46^ Further research should be performed to investigate whether gender differences in immune responses result in differential susceptibility of males and females to *P. aeruginosa* infection. Second, due to the unlikelihood of obtaining clinical *P. aeruginosa*‐infected lung tissue samples that meet strict control conditions, clinical samples from patients were not analyzed in this study. Third, current methods for single‐cell preparation are to some extent inadequate, often resulting in the loss of certain or rare cell types. The induction of transcriptional stress responses during tissue dissociation may also affect subsequent results.^47^ Finally, because inconsistency with RNA and protein levels may affect the analysis of scRNA‐seq results, we performed flow cytometry, immunofluorescence (IF), and mIHC to confirm the results of scRNA‐seq at the protein or cellular level, whereas throughput is still limited to a certain extent.

In conclusion, the results of this study provide insights into the transcriptional, molecular, and functional status of immune cells that occur within lung tissues following *P. aeruginosa* infection using scRNA‐seq and a variety of other techniques. We identified particular signatures of several new cell types during acute and chronic infections, vastly improving our understanding of infectious diseases. The main findings in our study may serve as valuable resources in other infectious mouse models and human infectious diseases, especially for understanding the roles that different host cell populations play during infection. These identified signaling pathways and key proteins in critical immune cells might have potential clinical implications.

## 4 MATERIALS AND METHODS

### 4.1 Bacterial strains

The *P. aeruginosa* reference strain PAO1, a gift from Dr. S. Lory (Harvard Medical School), was routinely cultured in Luria–Bertani broth with shaking (220 r.p.m.) at 37°C.

### 4.2 Mice

Eight‐week‐old female C57BL/6 mice were purchased from Beijing Huafukang Bioscience Co. Ltd. (Beijing, China) and housed in a specific‐pathogen‐free facility at the State Key Laboratory of Biotherapy, Sichuan University.

### 4.3 Mouse models

Acute and chronic infection model in mice were established according to previous study.^7^ The lung tissues were generated and divided into three parts. One part of the lung tissues was processed for H&E staining, and an independent assessment was conducted according to the scoring system of the American Thoracic Society.^48^ Other lung tissues were digested into single‐cell suspensions for scRNA‐seq and bulk RNA‐seq.

### 4.4 Fluorescence‐activated Cell Sorting (FACs) and reinfusion of IMs and in chronic infection model in mice

IMs were depleted by the CSF1r inhibitor PLX5622 (MCE, HY‐114153). C57BL/6 female mice were chowed by PLX5622 for 3 days. IMs were or not reinfused into the mice. Then, all mice were chronically infected for a week. Detection of survival curve, colony‐forming unit (CFU), H&E, and CD4^+^ and CD8^+^ T cells were conducted after a week.

### 4.5 Lung tissue dissociation and single‐cell suspension preparation

Freshly harvested control, acutely infected (12 h after infection) and chronically infected (10 days after infection) mouse lungs were digested with a Lung Dissociation Kit (MACs, 130‐095‐927), and single‐cell suspensions were generated according to the manufacturer's protocol.

### 4.6 Single‐cell RNA sequencing

The single‐cell suspension was loaded into Chromium microfluidic chips. Sequencing libraries were constructed with reagents from a Chromium Single Cell 3’ reagent kit (10X Genomics) according to the manufacturer's protocol. The count matrices were generated by Cell Ranger Single Cell Software (v3.1.0). Further analyses were performed in R using Seurat (v3.1), Monocle2, GSEA, CellChat, etc.

### 4.7 Generation and analysis of scRNA‐seq

The six samples include Ctrl1, Ctrl2, Acute1, Acute2, Chronic1, and Chronic2. The sequencing saturation of them are 71.5%, 76.6%, 80.9%, 75.1%, 69%, and 71.6%. Their number of reads are 456,488,848; 521,856,623; 488,500,735; 418,427,751; 472,993,752; and 473,312,146. The total genes detected of the samples are 17,620; 18,049; 17,423; 17,172; 18,016; and 17,894. And their number of cells are 10,818; 10,276; 10,172; 9,750; 13,352; and 13,243. The chemistry used in this study was Single Cell 3’ (v3). Based on the detailed information, we merged and integrated the metrics of six samples together after removing genes expressed in fewer than 150 UMIs and cells that had more than 10% mitochondrial genes. We filtered the ribosomal and red cell genes with Rp‐ and Hbb‐ initially expressed. Then, principal component analysis and uniform manifold approximation and projection dimension reduction (resolution = 0.5) with 30 principal components were performed. The RNA slot of the merged object was used as an input for Harmony.^49^, ^50^ The Louvain algorithm was used for community detection. We annotated cell clusters based on canonical marker gene and previous reports. Then, we only analyzed the immune cells following dimension reduction clustering of T and NK cells, B cells, and myeloid cells according to the initial annotation.

### 4.8 Pseudotime analysis

Filtered and merged datasets were imported into Monocle2 and generated a Seurat object. For unbiased trajectory and pseudotime analysis of the CD4^+^ and CD8^+^ T‐cell subpopulations, trajectory analysis was performed as previously described.^51^, ^52^

### 4.9 Enrichment analysis of marker genes

We applied the clusterProfiler R package to analyze the statistical enrichment of marker genes in KEGG pathways. We also used GSEA to analyze differentially expressed genes following obtaining enriched KEGG pathways. In addition, specific exploration of the gene lists for each comparison was also performed to identify relevant themes for genes whose function is described in the text (e.g., gene signatures of IM1). For this purpose, we only considered genes whose fold change was absolute >1.0 and whose *p*‐value < 0.05.

### 4.10 Analysis of cell–cell interactions

Cell–cell interactions based on the expression of known ligand–receptor pairs in different cell types were calculated using the CellChat R package as previously described.^53^ We applied the R package according to its standard analysis process and generated information with statistical significance. We used all cells in the three groups and the *p*‐value threshold included three sections: *p* < 0.01, 0.01 < *p* < 0.05, and *p* > 0.05.

### 4.11 Bulk RNA sequencing

Total RNA was extracted from cell suspension from the six samples. The samples were constructed into libraries through NEBNext Ultra RNA Library Prep Kit for Illumina (NEB, E7530L) following manufacturer's manuals. Sequencing data were generated on Illumina HiSeq 2000 platform by Novogene. The further analysis like differential expressed genes, GO, and KEGG was conducted by clusterProfiler R package.

### 4.12 Lung tissue dissociation and flow cytometry

After the single‐cell suspensions were generated and red blood cells were lysed using red blood cell lysis buffer (Beyotime, C3702), the cells were filtered through a 40 μm cell strainer (Falcon, 352340). Cells were counted and stained with the following fluorochrome‐conjugated antibodies from Biolegend: CD45 (103115, 1:100), CD3 (100236, 1:100), CD4 (100405, 1:100), CD8a (1000705, 1:100), CD19 (152407, 1:100), CCR7 (120107, 1:100), CD62L (104417, 1:100), CD11b (101207, 1:100), Ly‐6G (127617, 1:100), CD11c (117307, 1:100), Mertk (151503, 1:100), and Siglec‐F (155523, 1:100). Multicolor analysis was performed on a BD FACSymphony analyzer, and data were analyzed with FlowJo (V4) software.

### 4.13 Fluorescent multiplex immunohistochemistry

Samples of lung tissues were extracted and fixed in 4% paraformaldehyde overnight, followed by paraffin embedding, sectioning (4 μm), deparaffinization, and rehydration, according to standard protocols. For AMs, antigen retrieval was performed in AR6 solution (Dako, AR600250ML) using the means microwave treatment (MWT). Primary rat antibody against Siglec‐F (eBioscience, 14‐1702‐82, 20 μg/ml) was incubated for 1 h at 37°C followed by detection using an anti‐rat antibody labeled with horseradish peroxidase (HRP). Visualization of Siglec‐F was accomplished using Opal 690 TSA Plus (Akoya, NEL810001KT, 1:100), after which the slides were placed in AR6 solution and heated using MWT. The slides were then incubated with primary rat antibody for CD11c (eBioscience, 14‐0114‐82, 1:50) for 1 h at 37°C, followed by detection using the anti‐rat antibody labeled by HRP (ZSGB‐Bio, SP‐9001). CD11c was visualized using Opal 520 TSA Plus (Akoya, NEL810001KT, 1:100). For IMs, antigen retrieval was performed in AR6 solution using MWT. A primary rat antibody recognizing Mertk (eBioscience, 14‐5751‐82, 1:200) was incubated for 1 h at 37°C followed by detection using the anti‐rat antibody labeled with HRP. Visualization of Mertk was accomplished using Opal 690 TSA Plus (1:100), after which the slides were placed in AR6 solution and heated using MWT. The slides were then incubated a primary rat antibody recognizing CD11b (eBioscience, 14‐0112‐82, 1:50) for 1 h at 37°C, followed by detection using an anti‐rat antibody labeled by HRP. CD11b was visualized using Opal 520 TSA Plus (1:100). For Cxcl2^+^ B cells, antigen retrieval was performed in AR6 solution using the MWT. A primary rat antibody recognizing CD19 (eBioscience, 14‐0193‐82, 1:100) was incubated for 1 h at 37°C followed by detection using an anti‐rat antibody labeled by HRP. Visualization of CD19 was accomplished using Opal 690 TSA Plus (1:100), after which the slides were placed in AR6 solution and heated using MWT. The slides were then incubated with a primary rat antibody recognizing Cxcl2 (eBioscience, 701126, 1:100) for 1 h at 37°C, followed by detection using an anti‐rat antibody labeled by HRP. Cxcl2 was visualized using Opal 520 TSA Plus (1:100). Nuclei were subsequently stained with 4′,6‐diamidino‐2‐phenylindole (DAPI) (blue, Akoya, NEL810001KT, 1:50), and sections were cover slipped using anti‐fluorescence quenching agent. The slides were scanned using a Mantra System (PerkinElmer).

### 4.14 Immunofluorescence

Lung tissues were fixed in 4% paraformaldehyde overnight at room temperature, followed by paraffin embedding, sectioning (4 μm), deparaffinization, and rehydration. Immunodetection of neutrophils were performed on sections with the primary antibody goat anti‐human/mouse myeloperoxidase antigen affinity‐purified polyclonal antibody (RD system, AF3667) at 15 μg/ml for 3 h at room temperature. Then, cells were stained using NorthernLights 557‐conjugated anti‐goat immunoglobulin G secondary antibody (red, RD system, NL001) and counterstained with DAPI (blue, Beyotime, C1005).

### 4.15 Statistical analysis

Comparisons between two different groups were performed by unpaired two‐tailed Student's *t*‐test; comparisons between more than two groups were performed by one‐way analysis of variance (with Dunnett's multiple comparisons test). All *p*‐values less than 0.05 were considered significant. *p*‐Values are indicated on plots and in figure legends (^*^ *p* < 0.05, ^**^ *p* < 0.01, ^***^ *p* < 0.001, ^****^ *p* < 0.0001). Statistical analysis was performed with GraphPad Prism 8.

## AUTHOR CONTRIBUTIONS

T.L. and X.Z. initiated and conceived the project. X.H. and M.W. designed and performed the experiments. X.H. performed computational analyses. T.M., Y.G.Z., Y.X.Z., R.W., and C.Z. assisted with mouse experiments and prepared mouse samples. Y.R., H.L., Q.L., H.L., and X.S. assisted with the flow cytometry experiments. Y.Y., T.W., and M.T. assisted with mIHC experiments. X.H., J.L., T.L., X.G., X.L., and X.Z. wrote the manuscript with extensive input from all authors. All authors have read and approved the final manuscript.

## CONFLICT OF INTEREST

The authors declare that they have no conflicts of interest.

## ETHICS STATEMENT

All animal experiments were reviewed and approved by the Ethics Committee of the State Key Laboratory of Biotherapy, West China Hospital, Sichuan University (No. 20140104) and carried out in compliance with institutional guidelines concerning animal use and care of Sichuan University.

## Supporting information

Supporting Information

## Data Availability

Raw and processed single‐cell RNA sequencing and bulk RNA sequencing datasets have been deposited in the NCBI GEO database under accession GSE192890 and can be accessed via the following link: https://www.ncbi.nlm.nih.gov/geo/query/acc.cgi?acc=GSE192890. The data that support the findings of this study are available from the corresponding author upon reasonable request.
