# Supplementary material for: Single‐cell transcriptomics reveals distinct cell response between acute and chronic pulmonary infection of Pseudomonas aeruginosa
Source: MedComm (2020). 2022 Dec 8;3(4):e193. doi: 10.1002/mco2.193 (PMC9732387; doi:10.1002/mco2.193)
Supplement: Supplementary file 1 — Supporting Information [file MCO2-3-e193-s001.pdf]

Single-cell transcriptomics reveals distinct cell response between acute and chronic pulmonary infection of *Pseudomonas aeruginosa*

Xueli Hu[1#], Mingbo Wu[1#], Teng Ma[1], Yige Zhang[1], Chaoyu Zou[1], Ruihuan Wang[1], Yongxin Zhang[1], Yuan Ren[1,2], Qianqian Li[1], Huan Liu[1], Heyue Li[1], Taolin Wang[1], Xiaolong Sun[1], Yang Yang[1], Miao Tang[1], Xuefeng Li[4], Jing Li[2], Xiang Gao[3], Taiwen Li[2*], Xikun Zhou[1*]

[1]State Key Laboratory of Biotherapy and Cancer Center, West China Hospital, Sichuan University and Collaborative Innovation Center for Biotherapy, Chengdu 610041, China.

[2]State Key Laboratory of Oral Diseases, National Clinical Research Center for Oral Diseases, Chinese Academy of Medical Sciences Research Unit of Oral Carcinogenesis and Management, West China Hospital of Stomatology, Sichuan University, Chengdu, China.

[3]Department of Neurosurgery and Institute of Neurosurgery, State Key Laboratory of Biotherapy and Cancer Center, West China Hospital, West China Medical School, Sichuan University and Collaborative Innovation Center for Biotherapy, Chengdu, 610041, PR China.

[4]Department of Radiation Oncology, The University of Texas MD Anderson Cancer Center, Houston, TX, USA.

#These authors contributed equally: Xueli Hu and Mingbo Wu.

[*]To whom correspondence should be addressed:

Xikun Zhou

State Key Laboratory of Biotherapy and Cancer Center, West China Hospital, Sichuan University and Collaborative Innovation Center for Biotherapy, Chengdu, 610041, China.

E-mail: xikunzhou@scu.edu.cn

Taiwen Li

State Key Laboratory of Oral Diseases, National Clinical Research Center for Oral Diseases, Chinese Academy of Medical Sciences Research Unit of Oral Carcinogenesis and Management, West China Hospital of Stomatology, Sichuan University, Chengdu, 610041, China.

E-mail: litaiwen@scu.edu.cn

Supplemental Figure and Figure Legends

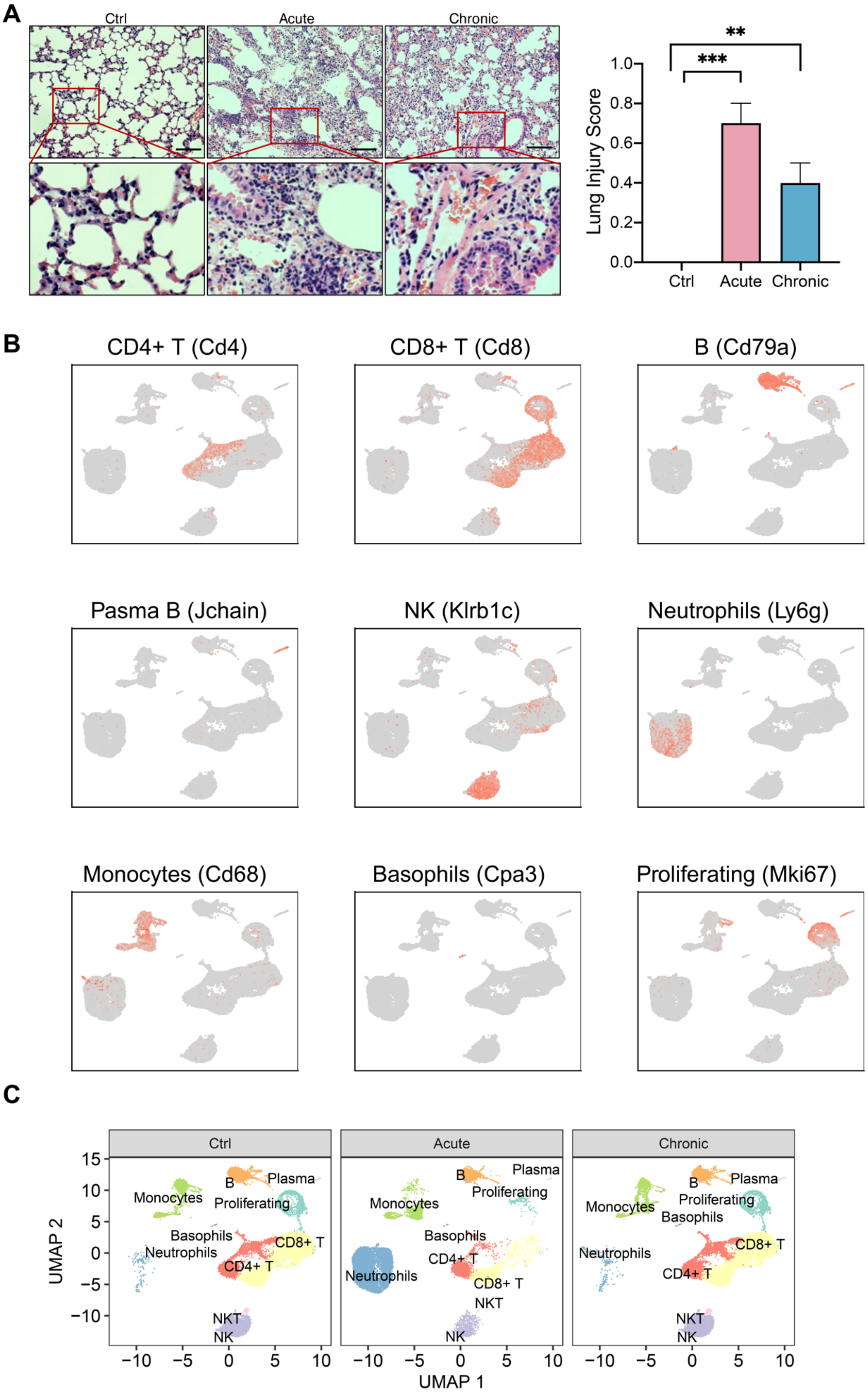

Supplemental Fig. 1 | Confirmation of Infection States and Specific Immune Cell Cluster Definition. **A**, Lungs embedded in formalin were evaluated by H&E staining. Images are representative of three independent replicates. Scale bar, 100 μm. Bars represent Mean ± SD. Significant differences were designated by using ANOVA followed by Dunnett's multiple comparisons test. $^{*}P < 0.05$, $^{**}P < 0.01$, $^{***}P < 0.001$. All data are shown representative of 2 separate experiments. **B**, UMAPs depicting the distribution of immune cell types and states in the three groups (split-view). **C**, UMAP plots depicting the markers used to identify the different immune cell types. Expression levels are color coded, tomato indicates a higher expression level, and gray indicates a lower expression level.

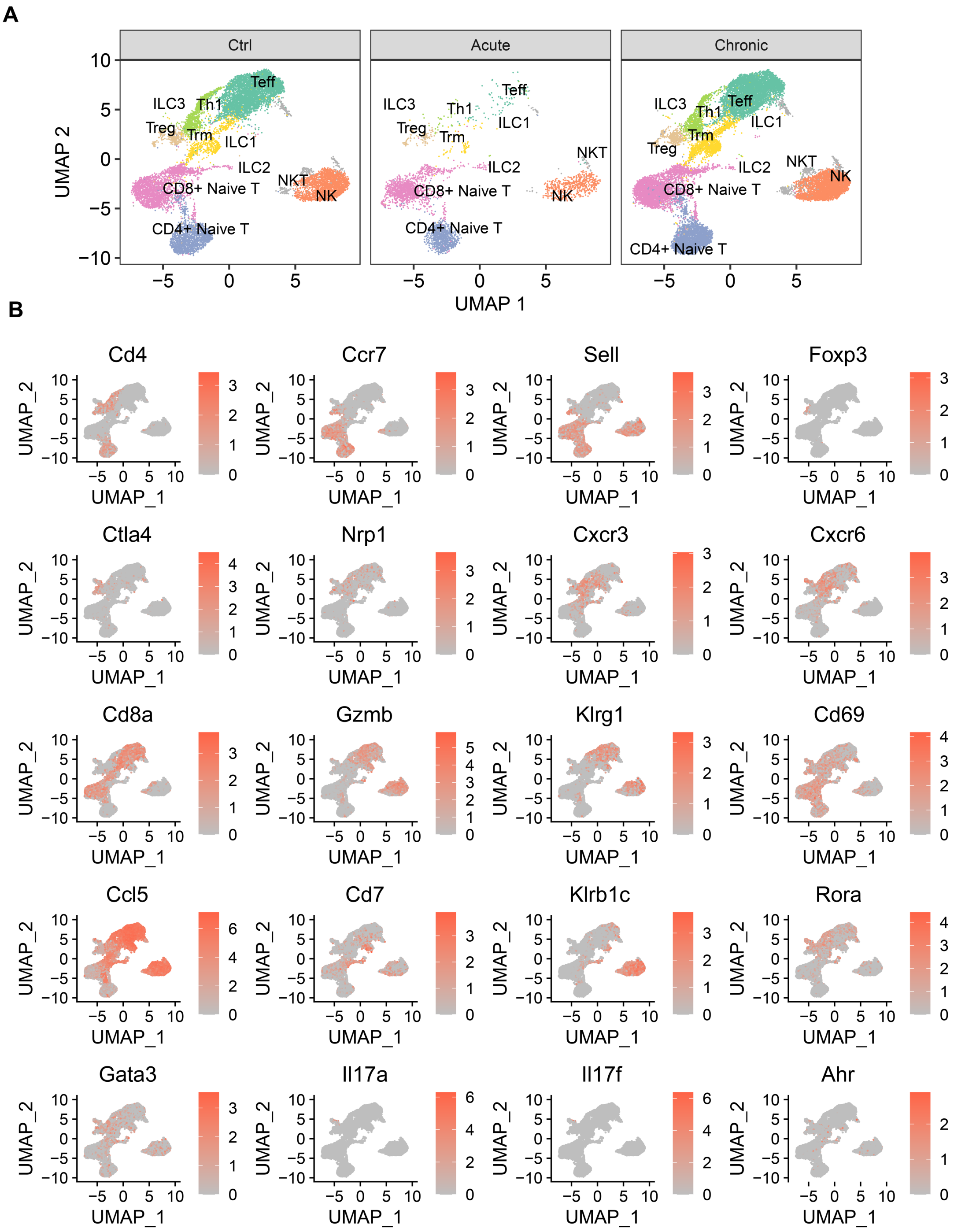

Supplemental Fig. 2 | Distribution of T and NK Cell Subtypes in the Three Groups and Markers Used to Identify. A, UMAPs depicting the distribution of T and NK cell subtypes and states in the three groups (split-view). **B**, UMAP plots depicting the markers used to identify the different T and NK cell subtypes. Expression levels are color coded, orange red indicates a higher expression level, and gray indicates a lower expression level.

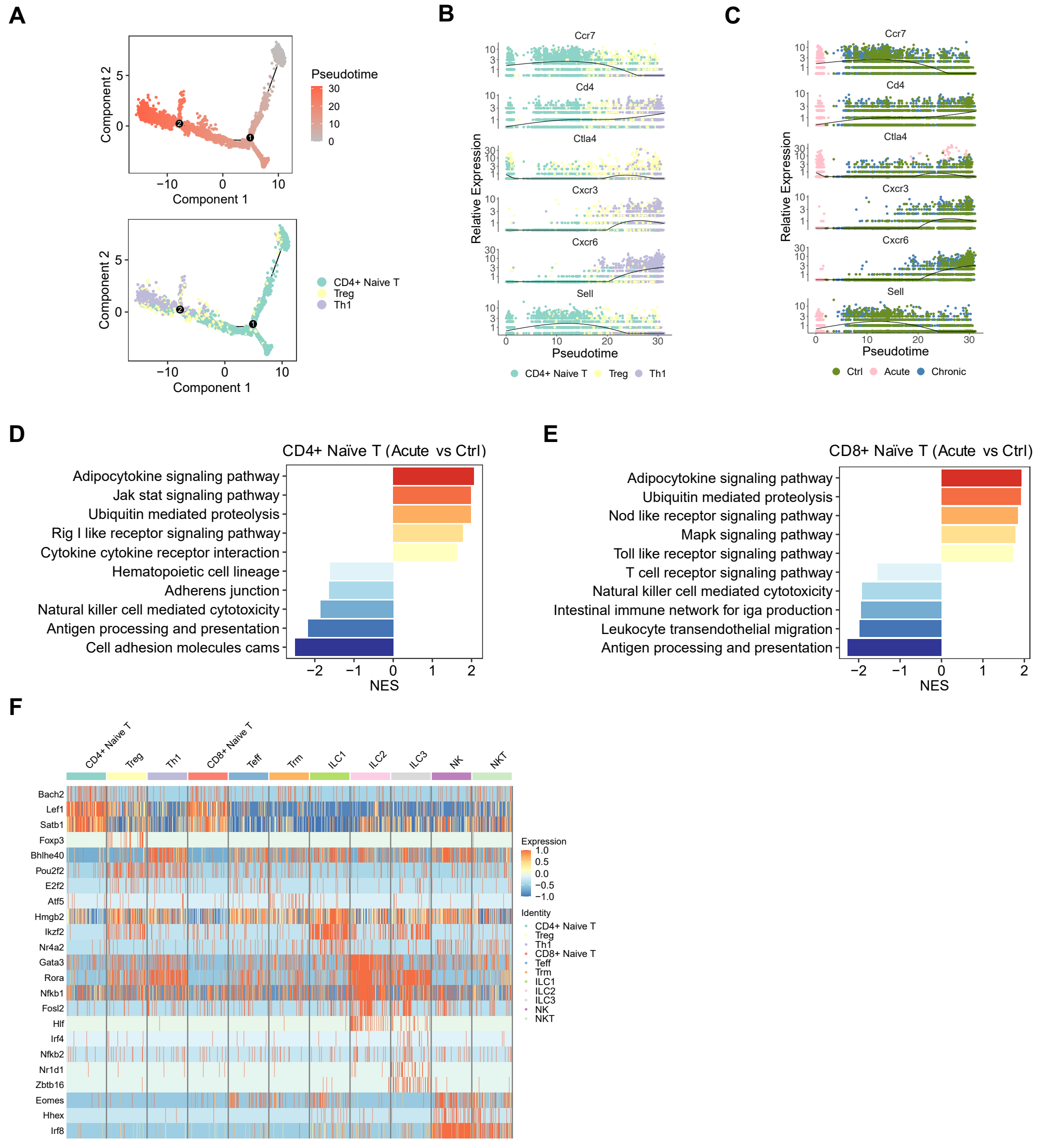

Supplemental Fig. 3 | Pseudotime Trajectories for CD4^{+} T Cells and Gene Signatures of Lymphocyte Subsets with Highly Dynamic Changes. A, Pseudotime trajectories for CD4^{+} T cells (CD4^{+} naïve T, Th1, Treg) showed that different branches of CD4^{+} naïve T cells differentiated into Th1 and Treg cells. **B**, Profiling of marker genes along these trajectories to confirm their functional annotation: Cd4, Ccr7, Sell, Cxcr3, Cxcr6, and Ctla4 for the lineage of CD4^{+} T cells. **C**, Density plots reflecting the marker genes along these trajectories stratified for the three groups: Ctrl, Acute and Chronic. **D**, **E**, Gene set enrichment analysis (GSEA) of differentially expressed genes between the Acute and Ctrl groups in CD4^{+} naïve T cells (**D**) and CD8^{+} naïve T cells (**E**), showing several pathways enriched in the Acute and Ctrl groups. The right bars indicate the pathways enriched in the Acute group, and the left bars indicate the

pathways enriched in the Ctrl group. **F**, Heatmap of differentially expressed TFs of T and NK cell subsets.

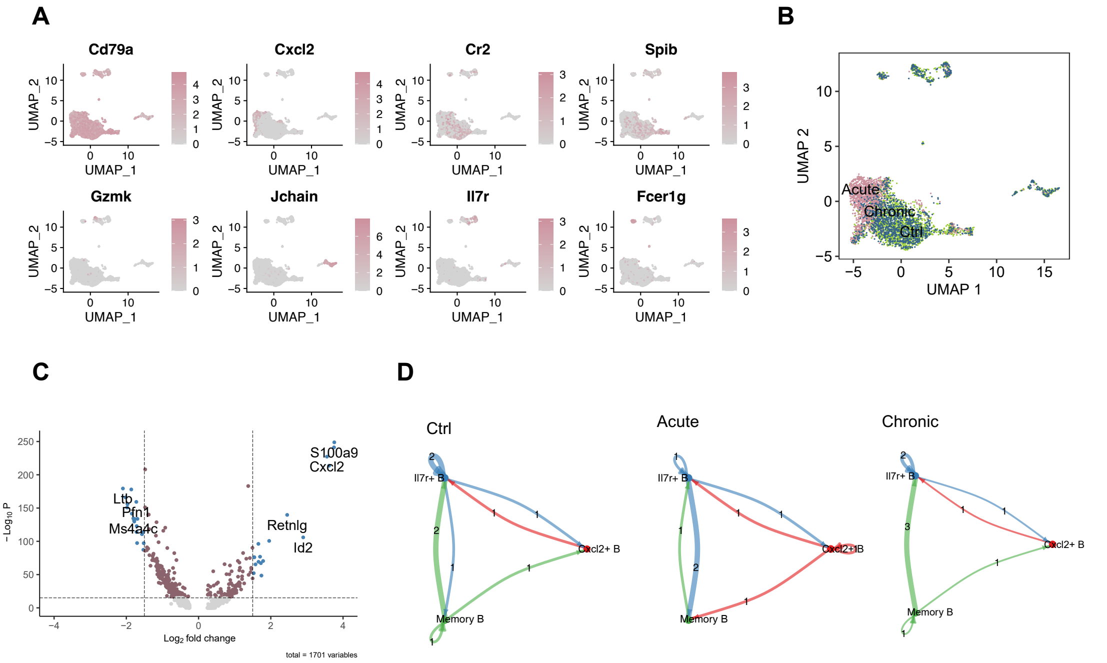

Supplemental Fig. 4 | Specific Identification of B Cell Subtypes and Dynamics of Cxcl2^{+} B Cells With Distinct Gene Signatures. A, UMAP plots depicting the markers used to identify the different B cell subtypes. Expression levels are color coded, pink indicates a higher expression level, and gray indicates a lower expression level. **B**, UMAP plots depicting the cell distribution of B cells in the three groups. **C**, Volcano plots of the differentially modulated genes of Cxcl2^{+} B cells in the Acute versus Ctrl groups. The x axis specifies the average log2-fold change (FC), and the y axis specifies the negative logarithm (base 10) of the adjusted P values. Left of the threshold value dots indicate genes whose expression is downregulated, right dots indicate genes whose expression is upregulated. Representative genes are labeled. **D**, Number of cell–cell interactions of Cxcl2^{+} B cells, Il7r^{+} B cells, and Memory B cells among the three groups.

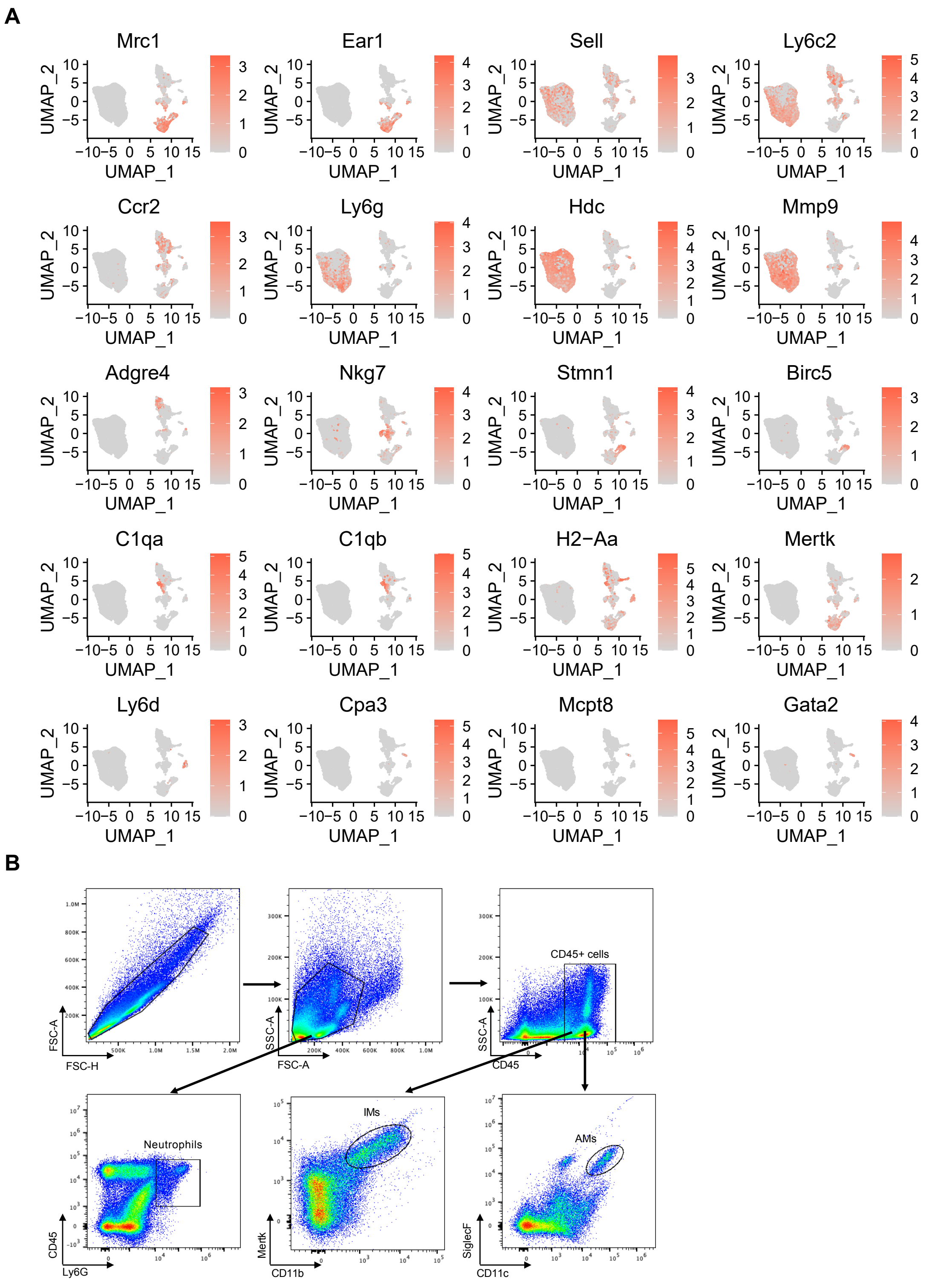

Supplemental 5 | Markers Used to Identify Myeloid Cell Subsets and Flow Cytometry Gating Strategy of Neutrophils, AMs, and IMs. A, UMAP plots depicting the markers used to identify the different myeloid cell subtypes. Expression levels are color coded, orange red indicates a higher expression level, and gray indicates a lower expression level. **B,** Flow cytometry gating strategy for myeloid cell subtypes, including neutrophils, AMs, and IMs.

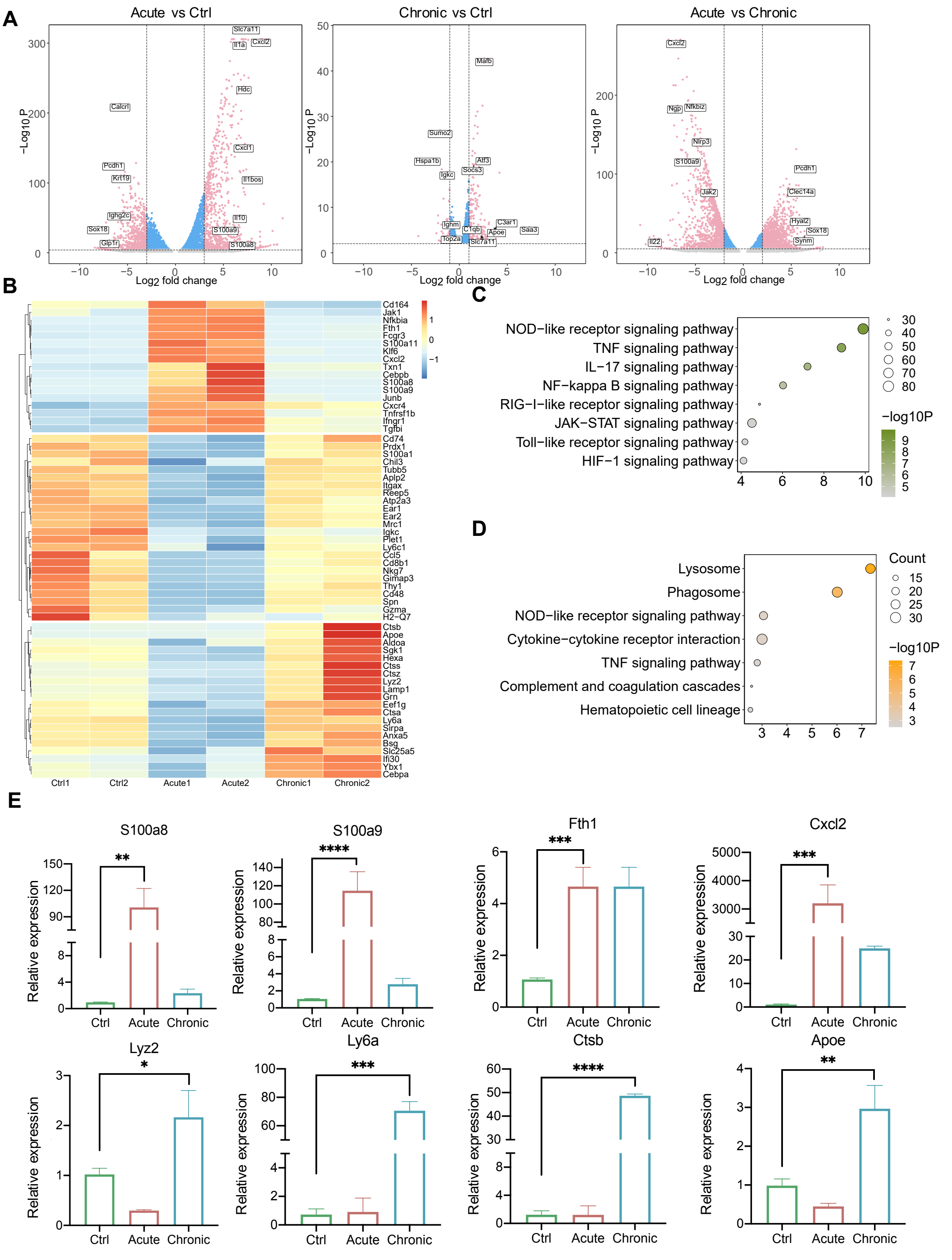

Supplemental 6 | Confirmation of the Overall Inflamed States by Analysis of Bulk-RNA Seq Results Among Three Groups. A, Volcano plots of the differentially modulated genes in the Acute versus Ctrl group, Chronic versus Ctrl group, and Chronic versus Acute group. The x axis specifies the average log2-fold change (FC), and the y axis specifies the negative logarithm (base 10) of the adjusted P values. Left of the threshold value dots indicate genes whose expression is downregulated, right dots indicate genes whose expression is upregulated.

Representative genes are labeled. **B**, Heatmap of differentially expressed genes among the six samples. **C**, **D**, Dotplot depicting the enriched signaling pathways in the Acute (**C**) and Chronic (**D**) groups. **E**, Verification of key genes in bulk RNA-seq by Q-PCR (n=3). Bars represent Mean ± SD. Significant differences were designated by using ANOVA followed by Dunnett's multiple comparisons test. *$P < 0.05$, **$P < 0.01$, ***$P < 0.001$. All data are shown representative of 2 separate experiments.

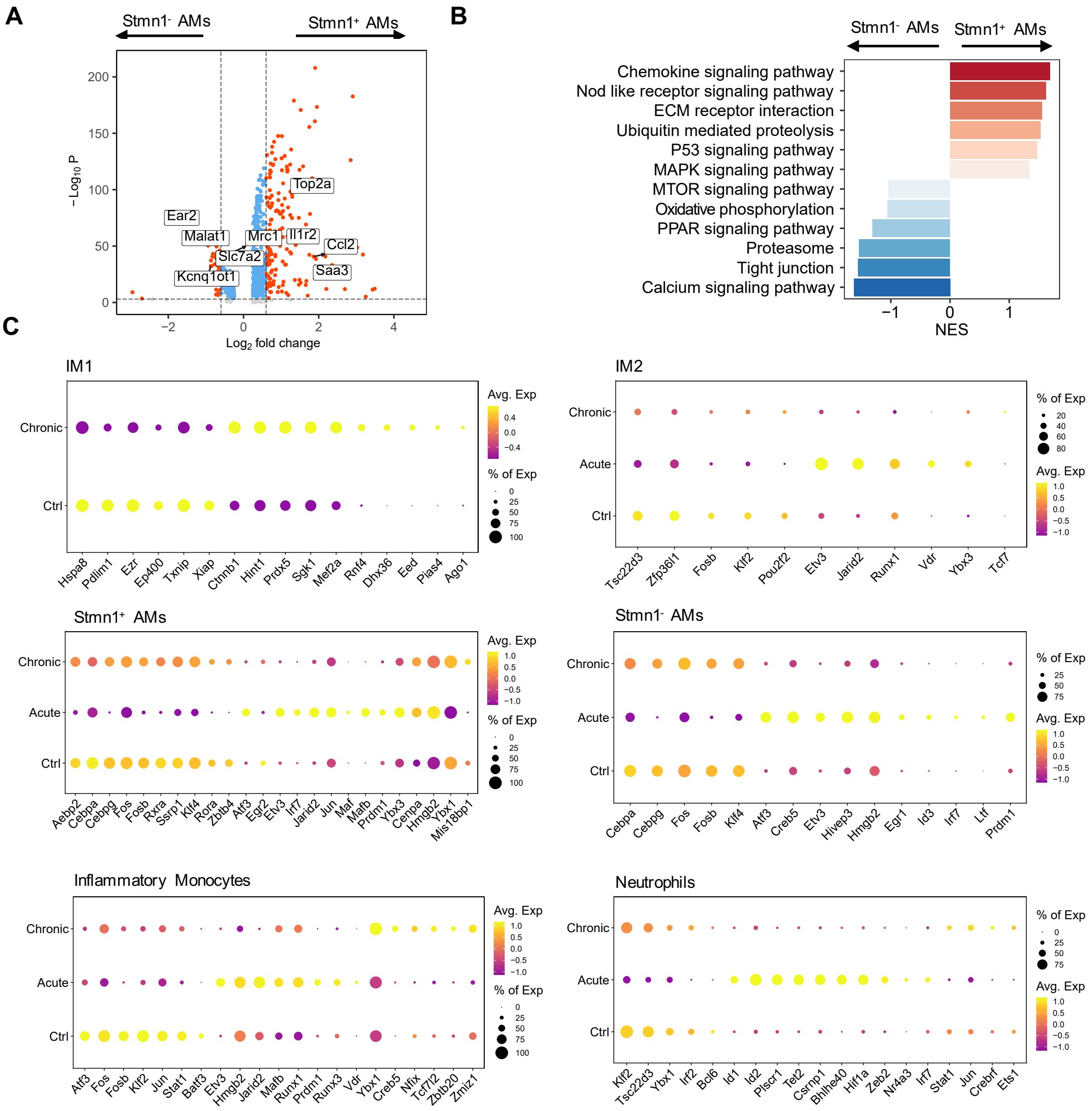

Supplemental Fig. 7 | Differentially Expressed Genes in Two Subtypes of AMs and Active TFs in Myeloid Cell Subsets. A, Volcano plots of the differentially modulated genes in Stmn1^{-} AMs versus Stmn1^{+} AMs. The x axis specifies the average log2-fold change (FC), and the y axis specifies the negative logarithm (base 10) of the adjusted P values. Left of the threshold value dots indicate genes whose expression is downregulated, right dots indicate genes whose expression is upregulated. Representative genes are labeled. **B**, Gene set enrichment analysis (GSEA) of differentially expressed genes between Stmn1^{-} AMs and Stmn1^{+} AMs showing several enriched pathways. The right bars indicate the pathways enriched in the Stmn1^{+} AMs, and the left bars indicate the pathways enriched in the Stmn1^{-} AMs. **C**, Dotpots depicting the differential TFs of IM1, IM2, Stmn1^{-} AMs, Stmn1^{+} AMs, inflammatory monocytes, and neutrophils among the three groups.

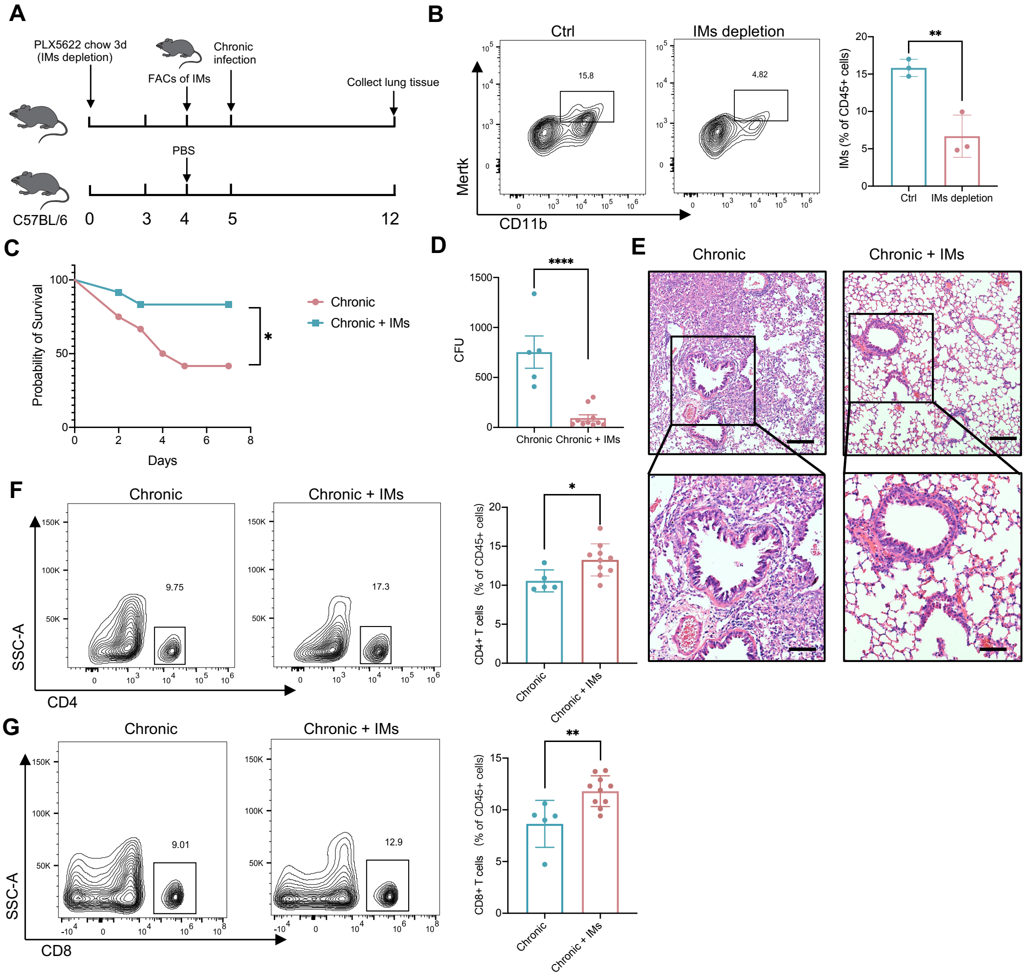

Supplemental Fig. 8 | Reinfusion of IMs Has a Protective Role for Host in Chronic Infection. A, Wild-type (WT) C57BL/6 female mice were divided into two groups, the one was chowed by PLX5622 for three days, reinfused by IMs which were generated by FACs, and then was chronically infected for a week. The other group was also chowed by PLX5622 for three days without reinfusion of IMs, then was also chronically infected for a week. **B,** Representative flow cytometry plots and quantification showing the effective depletion of IMs. **C**, The Kaplan–Meier survival curve for C57BL/6 female mice that were chronically infected with depletion of IMs (n = 12 per group) and chronically infected with reinfusion of IMs (n = 12 per group). Comparison of the survival curves was performed using the log-rank (Mantel–Cox) test. **D**, Lung residual CFU counts of the two groups. Bars represent Mean ± SD. Significant differences were designated by using t test. *P < 0.05, **P < 0.01, ***P < 0.001. **E,** Lungs embedded in formalin were evaluated by H&E staining. Images are representative of

three independent replicates. Scale bar, 100 μm. **F**, **G**, Representative flow cytometry plots and quantification showing the proportional changes in $CD4^+$ and $CD8^+$ T cells in the two groups (n=5, n=10). Bars represent Mean ± SD. Significant differences were designated by using t test. *P < 0.05, **P < 0.01, ***P < 0.001. All data are shown representative of 2 separate experiments.

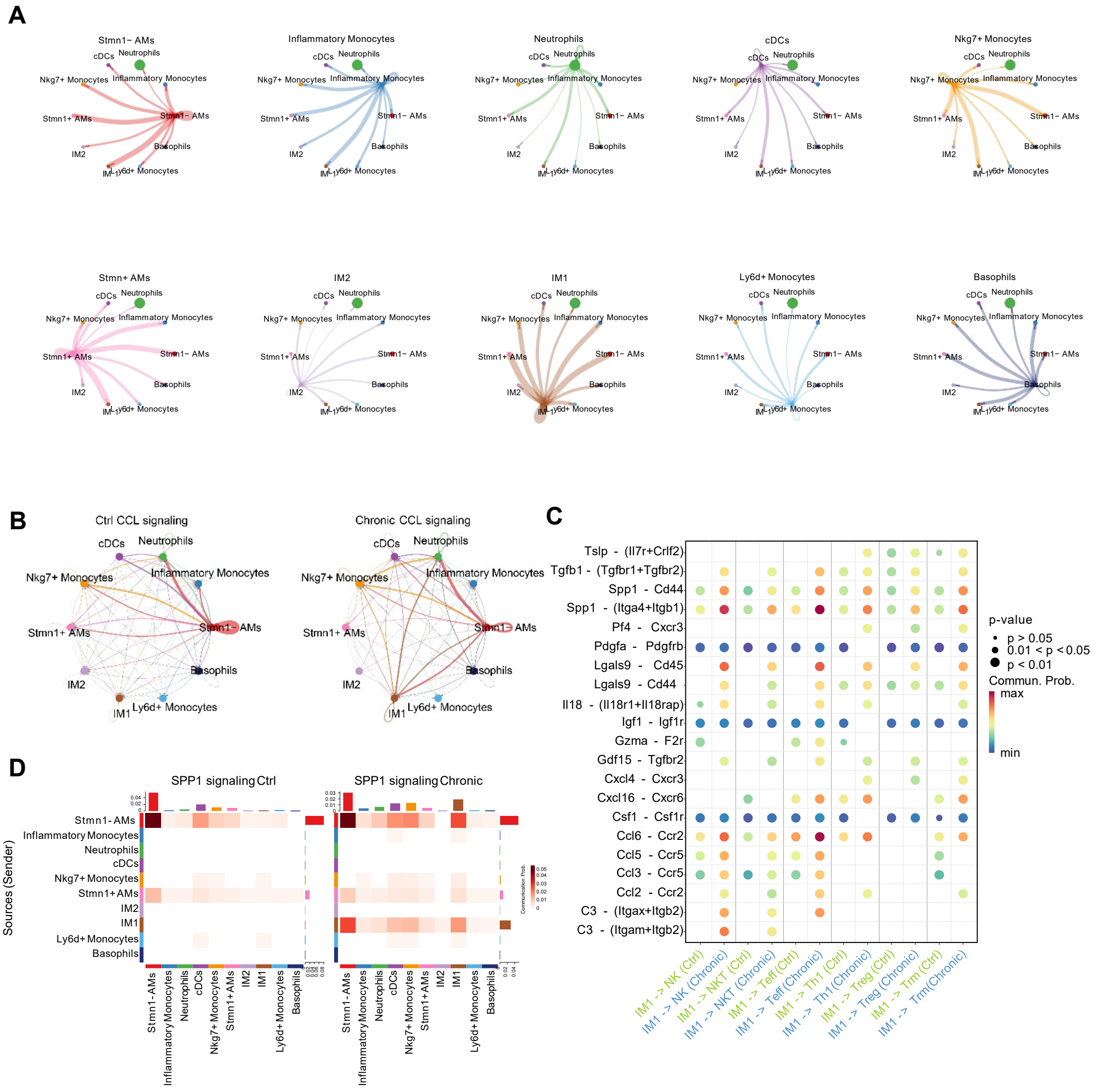

Supplemental Fig. 9 | Cell–Cell Interactions in Myeloid Cell Subtypes and Predictive Signaling Pathway Involved. A, Circle plots showing that myeloid cell subsets interact with other subclusters with different interaction strengths based on CellChat. **B**, Circle plots depicting how CCL signaling is involved in the cell–cell interaction between Stmn1^{-}AMs and other subclusters in the Chronic and Ctrl groups. **C**, Predictive ligand–receptor pairs involved in the cell–cell interactions between IM1, NK, NKT, Teff, Th1, Treg, and Trm in the Ctrl and Chronic groups. **D**, Heatmap depicting how SPP1 signaling is involved in the cell–cell interaction between Stmn1^{-}AMs and other subclusters in the Chronic and Ctrl groups.

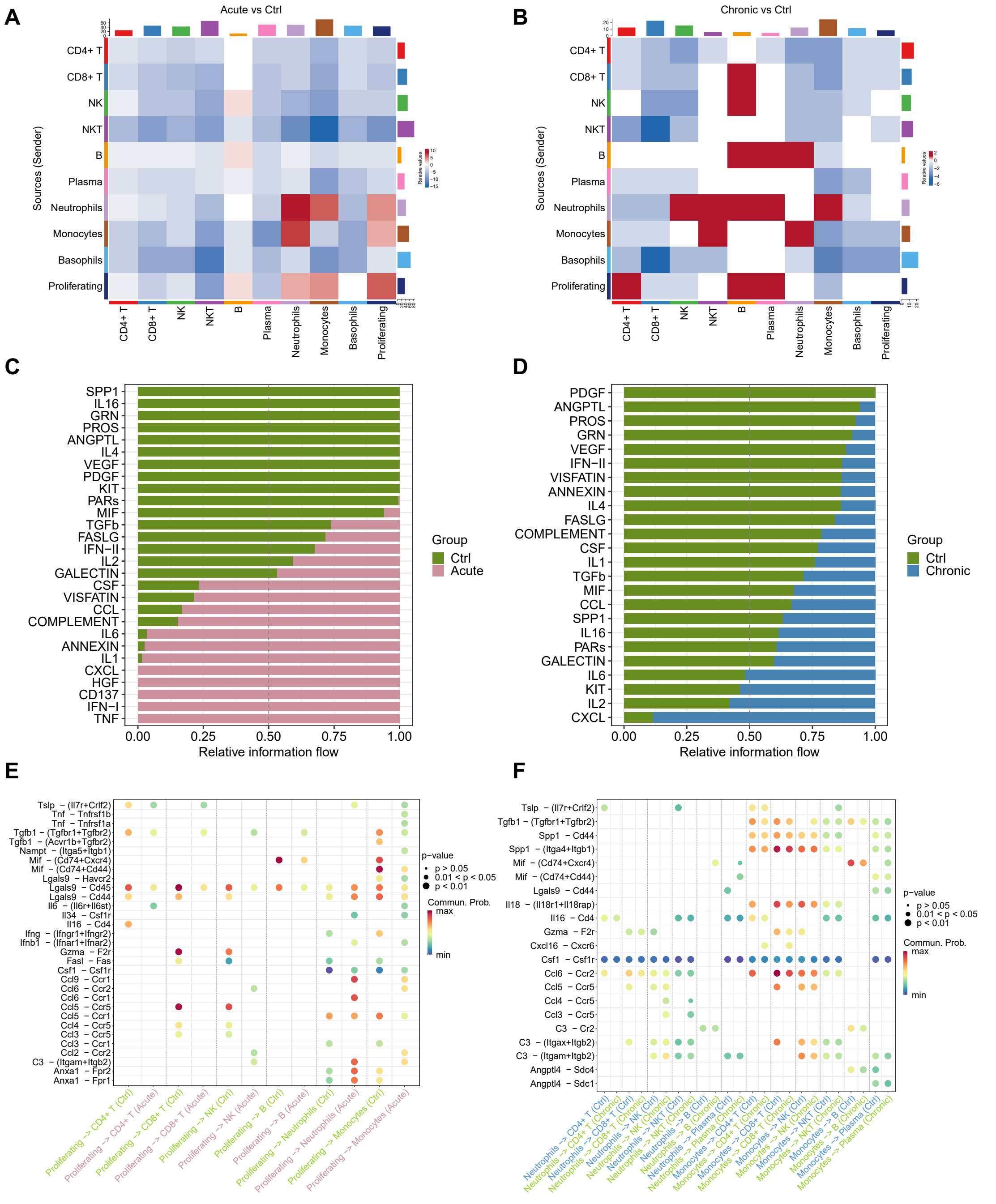

Supplemental Fig. 10 | Differential Cell–Cell Interactions of Immune Cell Subtypes Among Three Groups. A, **B**, Differences in the strength of interactions of immune subsets, comparing Acute vs Ctrl (**A**) and Chronic vs Ctrl (**B**). **C**, **D**, Overview of differential signaling pathways in cell–cell interactions of immune cell subsets, comparing Acute vs Ctrl (**C**) and Chronic vs Ctrl (**D**). **E**, **F**, Differential ligand–receptor pairs involved in specific cell–cell interactions of immune cell subsets, comparing Acute vs Ctrl (**E**) and Chronic vs Ctrl (**F**).
